# spaMGCN: a graph convolutional network with autoencoder for spatial domain identification using multi-scale adaptation

**DOI:** 10.1186/s13059-025-03637-z

**Published:** 2025-06-10

**Authors:** Tianjiao Zhang, Hongfei Zhang, Zhongqian Zhao, Saihong Shao, Yucai Jiang, Xiang Zhang, Guohua Wang

**Affiliations:** 1 College of Computer and Control Engineering, Northeast Forestry University, Harbin, 150040 China; 2 Faculty of Computing, Harbin Institute of Technology, Harbin, 150001 China

**Keywords:** Spatial domain identification, Spatial multi-omics data, Discrete distribution spatial domain, Multi-source feature fusion

## Abstract

Spatial domain identification is crucial in spatial transcriptomics analysis. Existing methods excel with continuous and clustered distributions but struggle with discrete ones. We present spaMGCN, an innovative approach specifically designed for identifying spatial domains, especially in discrete tissue distributions. By integrating spatial transcriptomics and spatial epigenomic data through an autoencoder and a multi-scale adaptive graph convolutional network, spaMGCN outperforms baseline methods. Our evaluations demonstrate its effectiveness in recognizing discrete T cell zones in mouse spleens and follicular cells in human lymph nodes, as well as effectively distinguishing capsule structures from surrounding tissues.

**Supplementary Information:**

The online version contains supplementary material available at 10.1186/s13059-025-03637-z.

## Background

The advent of single-cell RNA sequencing (scRNA-seq) technology has enabled biologists to obtain genome-wide expression profile data at the single-cell level, thereby effectively analyzing heterogeneous cell populations within complex samples [1]. The rapid development of single-cell technologies provides a unique perspective for understanding cellular heterogeneity and dynamic changes in complex biological systems [2]. In recent years, emerging multi-omics sequencing technologies such as CITE-seq [3], SNARE-seq [4], and TEA-seq [5] have allowed researchers to capture multiple omics data, including transcriptomics and epigenomics, at the single-cell level, facilitating the analysis of cellular heterogeneity from various angles. However, in multicellular organisms, cells often form specific structures or tissue regions in an intertwined manner based on their spatial locations and functional similarities within a given tissue, which contribute to specific biological functions. For example, hepatocytes in the liver can form liver lobules, while cardiomyocytes in the heart are arranged into myocardial layers. Linking gene expression information to its spatial distribution is crucial for understanding the related functions of tissues. Current spatial transcriptomics technologies are primarily divided into in situ sequencing-based methods represented by 10 × Visium [6], Slide-seq [7], and Stereo-seq [8], and imaging-based methods represented by MERFISH [9] and seqFISH + [10]. Utilizing these spatial transcriptomics technologies allows for the acquisition of both transcriptomic data and spatial location information, making it possible to decipher tissue functions and cellular composition. However, these technologies capture only RNA-seq data, resulting in an inherent limitation of information scarcity. Consequently, spatial transcriptomics is expanding into spatial multi-omics, enabling biologists to capture expression patterns of different omics data simultaneously on a single tissue section [11]. Recently, several spatial multi-omics sequencing methods that integrate multi-omics sequencing technologies have been developed, such as DBiT-seq [12] and spatial-CITE-seq [13], which can simultaneously acquire gene expression profiles, protein data, and spatial location information. Additionally, spatial ATAC–RNA-seq and CUT&Tag-RNA-seq [14] can capture gene expression profiles, chromatin accessibility, and spatial location information. This additional omics information provides multiple perspectives for a deeper understanding and analysis of tissue heterogeneity and the functions of different tissue structures.

Identifying spatial domains is one of the key steps in analyzing spatial transcriptomics data and serves as the foundation for understanding tissue heterogeneity. Initially, some researchers utilized single-cell clustering methods such as Seurat [15] to perform cluster analysis on spatial transcriptomics data for identifying spatial domains, and employed spatialLIBD [16] and SpatialCPie [17] to visualize the results of these spatial domain delineations. However, these methods did not utilize spatial location information and overlooked the characteristic that most cells within the same spatial domain cluster together, resulting in poor performance in spatial domain identification. Subsequently, Zhao et al. [18] developed a fully Bayesian statistical method, BayesSpace, which leverages spatial neighborhood information to enhance the resolution of spatial transcriptomics data and perform clustering analysis. SC-MEB [19] models spatial transcriptomics data using a hidden Markov model to achieve spatial domain identification. However, these statistical probabilistic models often fail to extract relevant features that characterize tissue structures effectively when dealing with large-scale spatial transcriptomics datasets and require high-quality data, leading to suboptimal performance in spatial domain identification. SpaGCN [20] is the first deep learning method that employs graph convolutional networks to extract features from gene expression, spatial location, and morphological data, identifying spatial domains by generating undirected weighted graphs that capture spatial dependencies in data. GraphST [21] utilizes graph convolutional neural networks and self-supervised contrastive learning to optimize the representation of spatial information and enhance clustering performance. GAAEST [22] employs a graph attention network combined with a graph self-supervised contrastive learning mechanism, enhancing features through three different levels of spatial mutual information, thereby acquiring latent embeddings for accurate spatial domain delineation. Nevertheless, the amount of information contained within single-omics data is limited, especially in cases of complex tissue structures, making it challenging for spatial domain identification methods developed for transcriptomic data to achieve ideal results. The emergence of spatial multi-omics sequencing technologies breaks the traditional limitation of spatial transcriptomics, which only captures single-omics data, thereby leading to a shift in spatial domain identification methods from single transcriptomics data applications to the integration of spatial multi-omics data.

In the analysis of spatial multi-omics data, the information provided by different omics is complementary, allowing for multi-faceted characterization of tissue structures. By integrating spatial multi-omics data, it is possible to overcome the inherent limitations of single-omics data, leading to more accurate spatial domain identification. Recently, Long et al. [11] developed the first deep learning method, SpatialGlue, that utilizes spatial multi-omics data for spatial domain delineation. This model is a graph neural network with a dual attention mechanism that fuses spatial feature information with omics feature information through two separate attention modules, ultimately using the fused features from multiple omics to decode spatial domains. The SSGATE [23] method is capable of analyzing both single-cell multi-omics data and spatial multi-omics data. For single-cell multi-omics data, it constructs neighborhood graphs based on single-cell expression profiles, while for spatial multi-omics data, it builds neighborhood graphs based on spatial coordinates. It employs a graph attention network to extract features from different omics data and then uses the fused data to achieve single-cell clustering or spatial domain delineation.

Although both of these methods can leverage spatial multi-omics data to decode spatial domains, their performance still requires enhancement, and developing a more reliable method for identifying spatial domains using spatial multi-omics data remains an urgent issue to address. Additionally, we note that these methods, when identifying discrete distributions within the same spatial domain or subtle structures closely connected to other tissue layers, tend to overemphasize spatial information while neglecting the intrinsic attribute information of the spots themselves. This can result in these tissue structures being misclassified into different spatial domains, complicating the separation of adjacent tissue structures.

To this end, we propose a deep learning model named spaMGCN for spatial domain identification and downstream analysis of spatial multi-omics data, which combines an autoencoder with a multi-scale adaptive graph convolution module [24]. The autoencoder extracts attribute information from different omics data of each spot, allowing spatially discrete spots to be classified into the same spatial domain based on the attribute information contained in the various omics data. This attribute extraction enhances the clarity of the spatial domain boundaries. Leveraging the multi-scale adaptive convolution module, spaMGCN captures multi-order neighbor information of the spots and utilizes an adaptive attention mechanism to dynamically fuse this information to obtain multi-scale structural features. Additionally, through multiple skip connections, the model performs progressive fusion of the attribute information and structural information. Finally, we combine the multi-step fused representations from different omics to yield embedding representations that encompass features from multiple omics. Considering that most spatial domains are continuous and clustered, while also allowing interaction between the two omics feature extraction modules, we draw inspiration from Deep Graph Structural Infomax [25] to compute the binary cross-entropy loss between the reconstructed graph and the original graph structure, and calculate the contrastive learning loss similar to InfoNCE. This approach enhances the mutual information of adjacent nodes, ensuring that physically adjacent points are brought closer together in feature space, thereby enabling accurate spatial domain identification and elucidation of tissue heterogeneity. To validate the effectiveness of the spaMGCN method, we conducted experiments on 5-point-resolution spatial multi-omics datasets obtained from 10 × Genomics Visium, Stereo-CITE-seq, and SPOTS, as well as one single-cell resolution spatial multi-omics dataset. We compared spaMGCN with other advanced spatial domain identification methods, and the experimental results demonstrate that the spaMGCN model exhibits significant superiority in spatial domain identification performance. In addition, we also applied spaMGCN to a series of spatial transcriptomics datasets to validate its wide applicability.

## Results

### Overview of spaMGCN

**Fig. 1:**
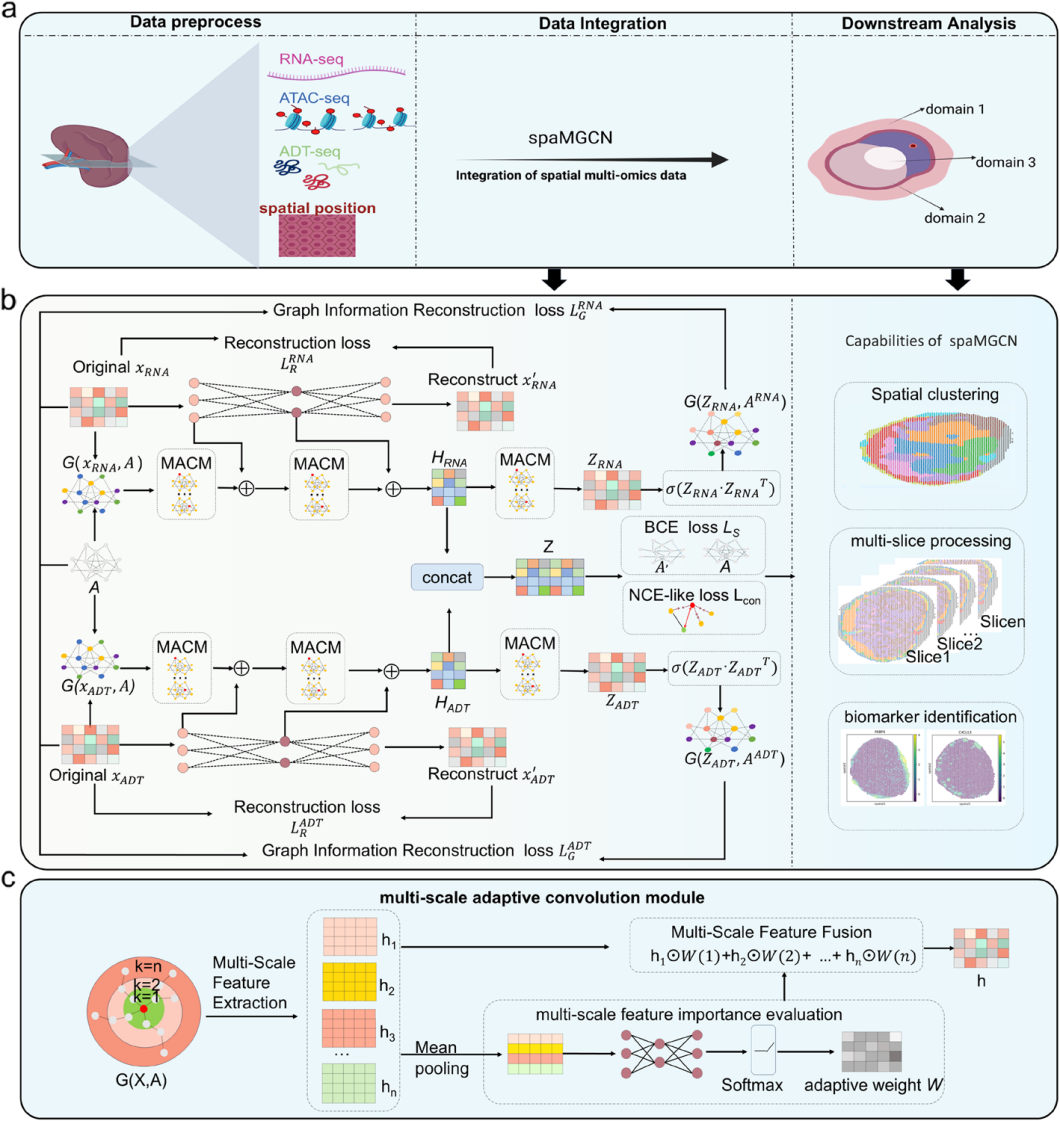
Overall framework diagram of the spaMGCN model. **a** Spatial domain identification and downstream analysis utilizing spatial multi-omics data. Created in https://BioRender.com. **b** The overall framework of spaMGCN. **c** Multi-scale adaptive graph convolution module for extracting multi-scale structural information

The framework of spaMGCN is illustrated in Fig. 1. It consists of two parallel feature extraction modules and a multi-omics feature fusion module. The two feature extraction modules are designed to extract multi-source fused features from gene expression profiles and other omics data, using the same model architecture. After preprocessing the different omics data and adjacency relationships, we input the various omics data into the corresponding autoencoder modules to extract attribute features of different spots. The different omics data and the spatial neighborhood graph are fed into the respective graph encoders composed of multi-scale adaptive graph convolution modules to extract structural features from the various data types. To ensure that the extraction of structural features does not result in the loss of the intrinsic attribute features of the spots—crucial for the clear identification of discrete spatial domains—we fuse the spot-specific attribute features extracted from different layers of the autoencoders with the structural features obtained by the graph encoders, which take into account the attribute features of neighboring spots. This incorporation ensures that the model considers both the spatial positions of the spots and their intrinsic attribute information, maintaining the compatibility of spaMGCN for recognizing continuous and discrete spatial domains. Subsequently, we merge the multi-source fused features from different omics again to obtain a fused feature representation that encompasses various omics information. Finally, we utilize the Kmeans algorithm to perform clustering analysis on the spatial multi-omics fused features to identify spatial patterns of different tissues.

### Comparison of clustering performance between spaMGCN and existing methods

We first conducted experiments on a simulated dataset [11], as shown in Additional file 1: Fig. S1. From the spatial domain identification results, we observed that all methods utilizing spatial multi-omics data could clearly reconstruct the structure of Domain 0 (the simulated cross-shaped region). In contrast, GraphST and GAAEST failed to restore the cross-shaped pattern, even though GraphST achieved an ARI of 0.989. Due to the lack of additional omics information, these methods were unable to fully identify the structure of Domain 0. This demonstrates the effectiveness and rationale of employing spatial multi-omics data for spatial domain identification.

**Fig. 2:**
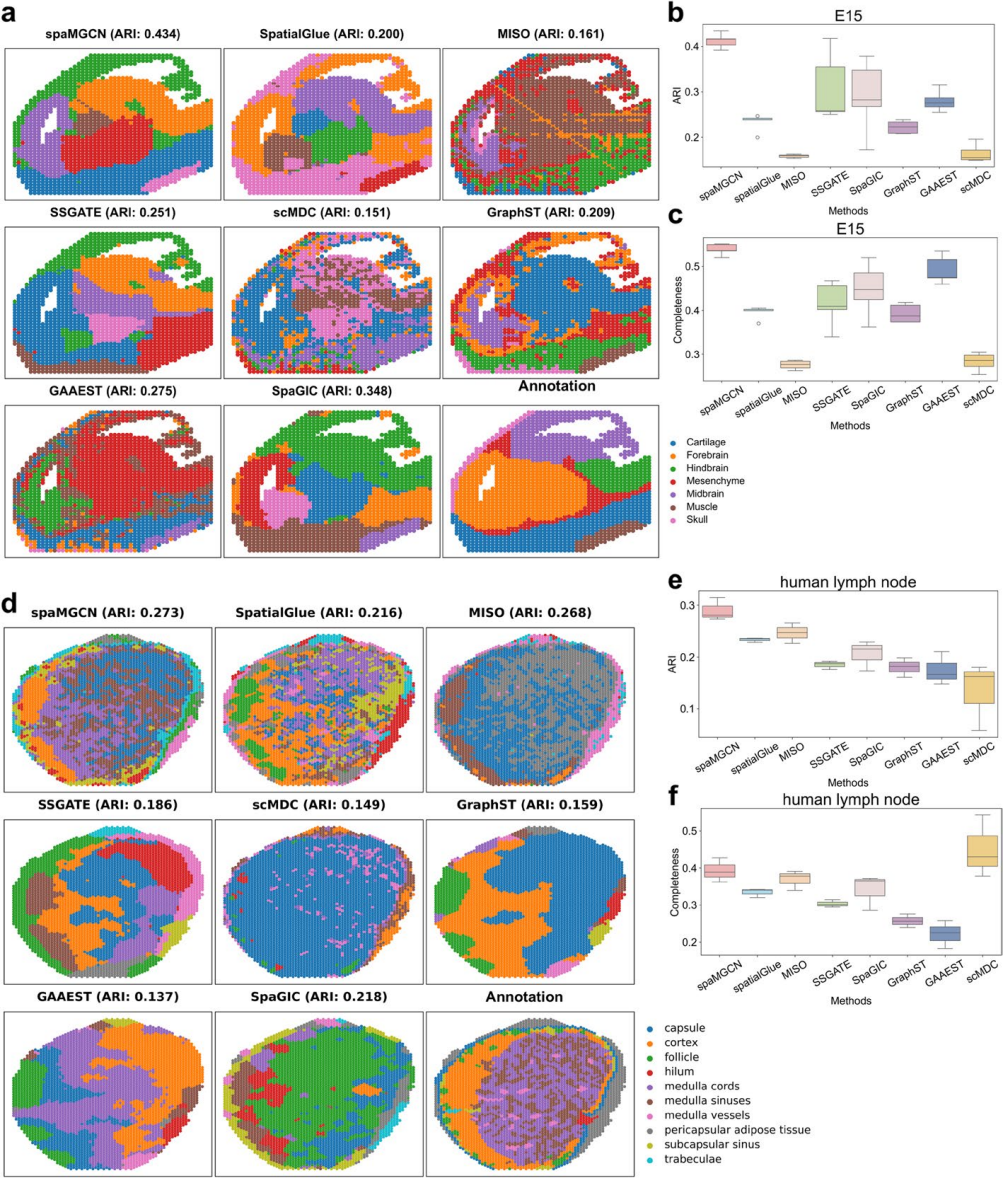
Spatial domain identification performance of different methods on four datasets. **a** Visualization of spatial domain prediction results from the spaMGCN method compared to other methods on the E15 dataset. **b** ARI metrics of different methods on the E15 dataset. **c** Completeness metrics of different methods on the E15 dataset. **d** Visualization of spatial domain prediction results from the spaMGCN method compared to other methods on the human lymph node S1 dataset. **e** ARI metrics of different methods on the human lymph node S1 dataset. **f** Completeness metrics of different methods on the human lymph node S1 dataset

Next, we conducted experiments on the E15 dataset, a 15-day mouse brain measured by MISAR-seq technology. As shown in Fig. 2a, spatial domain segmentation results demonstrate that spaMGCN clearly separates boundaries among three adjacent tissues (Skull, Cartilage, and Forebrain). Figure 2b, c, and Additional file 1: Fig. S2 reveal that spaMGCN outperforms seven baseline methods across six clustering metrics, with its average ARI being 10.48% higher than the second-best method, SSGATE.

Subsequently, to further validate spaMGCN’s performance, we tested it on three human lymph node spatial multi-omics datasets generated by 10 × Genomics Visium. Figure 2d and Additional file 1: Fig. S3-S4 visualize the spatial clustering results across three datasets. Figure 2e, f, and Additional file 1: Fig. S5 show that spaMGCN achieves optimal results on all metrics except Completeness Score. Visualization of spatial domains on slice S1 highlights key differences: unlike the E15 dataset, lymph node structures exhibit discrete patterns. Graph neural network-based methods (SSGATE, GraphST, GAAEST, SpaGIC) produce oversmoothed boundaries due to overreliance on structural information, making them unsuitable for discrete tissues. Although MISO and scMDC preserve discreteness by fusing autoencoder-extracted multi-omics features, they neglect spatial relationships, which results in suboptimal performance. SpatialGlue implicitly utilizes spatial information via attribute graphs but still suffers from graph neural networks’ smoothing effects, leading to misclassification of adjacent heterogeneous regions.

The innovation of spaMGCN lies in its dynamic integration of attribute features (extracted by autoencoders) and multi-scale structural features (captured by the Multi-scale Adaptive Convolution Module, MACM), enabling robust performance on both discrete (lymph nodes) and continuous (E15) tissues. As illustrated in Fig. 2d, spaMGCN accurately distinguishes the medulla from the cortex and capsule, aligning with the anatomical “outer cortex-inner medulla” structure of lymph nodes. The effectiveness of multi-scale fusion will be further validated in parameter analysis and ablation studies.

Moreover, during the experimental process, we found that most methods employing spatial multi-omics data for spatial domain identification outperformed traditional methods that utilize single-omics data. This finding further confirms that utilizing spatial multi-omics data for spatial domain identification is both effective and necessary. This is attributed to the fact that multi-omics data provide richer information on tissue heterogeneity compared to single-omics data, enabling different perspectives on tissue structure. Therefore, integrating multi-omics data for analysis can aid in more accurately identifying spatial domains.

To assess the generalization ability of the spaMGCN model, we conducted experiments on two additional spatial multi-omics datasets: Mouse Thymus and Mouse Spleen, obtained using Stereo-CITE-seq and SPOTS technologies, respectively. As shown in Additional file 1: Fig. S6-S8, while MISO achieved the best performance on five out of six clustering metrics (excluding Homogeneity) for the spleen dataset, spaMGCN ranked second. This may be attributed to the spleen dataset’s inherent complexity, as evidenced by the low average ARI (< 0.2) across all methods. For the thymus dataset, annotations were derived from SpatialGlue’s clustering results followed by expert refinement based on differential protein and gene expression—not a gold-standard ground truth. Thus, we excluded SpatialGlue from comparative analyses on this dataset. After this exclusion, spaMGCN outperformed the six remaining methods on five of the six clustering metrics (excluding Homogeneity).

During the experimental setup, we applied a classic single-cell multi-omics clustering method, scDMC [26], for spatial domain identification. Its performance across all datasets was inferior to that of methods utilizing spatial multi-omics data for spatial domain identification. This is attributed to scDMC not leveraging spatial location information, a key factor in recognizing spatial domains. As a result, many spatially contiguous domains were fragmented, leading to a series of isolated points. However, compared to other methods that utilize single-omics data for spatial domain identification, the performance of scDMC varied across different datasets. We surmise that for spatially continuous tissues, spatial location information tends to be more critical. In contrast, when spatial domains are relatively discrete, multiple omics features often provide richer node attribute information, aiding in the recognition of discontinuous spatial domains that belong to the same tissue structure.

To prevent model overfitting and ensure generalizability across diverse datasets, we further evaluated the clustering performance of different methods using five-fold cross-validation on five spatial multi-omics datasets. As shown in Additional file 2: Table S1-S3, spaMGCN consistently achieved optimal performance across all five datasets under this rigorous validation framework. Overall, spaMGCN demonstrates outstanding performance in spatial domain identification across multiple spatial multi-omics datasets generated by different sequencing technologies, highlighting its excellent generalization capability.

To further validate the scalability of spaMGCN, we simplified its architecture by removing one parallel structure to enable processing of single-omics datasets. We then applied this modified version to three spatial transcriptomics datasets: human breast cancer, bronchial tumor, and mouse embryo E9.5, comparing its performance with three state-of-the-art methods for spatial domain identification (GraphST, GAAEST, and SpaGIC).

**Fig. 3:**
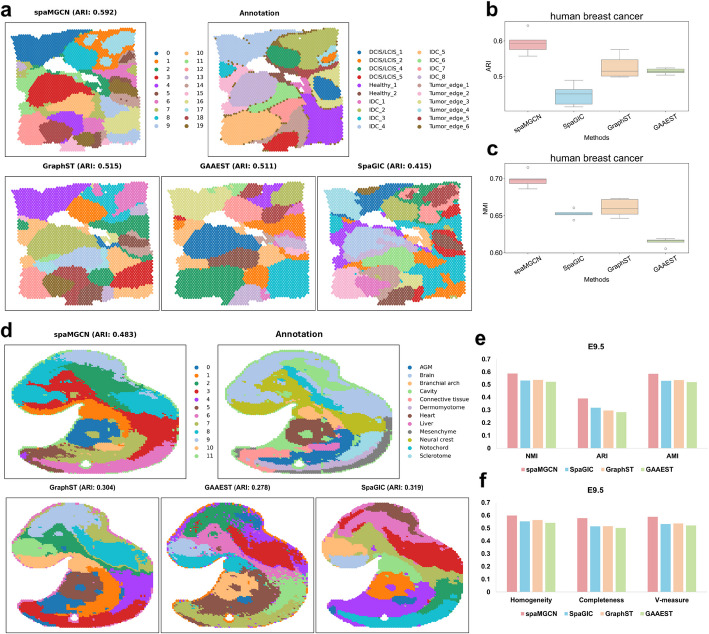
Visualization of spatial domain prediction results from the spaMGCN method compared to other methods on the HBC dataset and E9.5 dataset. **a** Visualization of spatial domain prediction results from the spaMGCN method compared to other methods on the HBC dataset. **b** ARI metrics of different methods on the HBC dataset. **c** NMI metrics of different methods on the E15 dataset. **d** Visualization of spatial domain prediction results from the spaMGCN method compared to other methods on the E9.5 dataset. **e** ARI metrics of different methods on the E9.5 dataset. **f** Completeness metrics of different methods on the E9.5 dataset

Beginning with the human breast cancer dataset, which presents significant challenges due to the highly heterogeneous nature of breast cancer that complicates the identification of distinct tumor regions, we found that accurate delineation of tumor boundaries is crucial for clinical applications. spaMGCN demonstrated superior performance, achieving a median ARI of 0.592 across five experiments, with a peak value of 0.64, and outperformed all three competing methods across six clustering metrics (Fig. 3b, c; Additional file 1: Fig. S9). Notably, spaMGCN was the only method that successfully identified both the IDC5 tumor region and its adjacent tissue region, Tumor_edge_4, while maintaining clear boundaries consistent with manual annotations. This precision can be attributed to spaMGCN’s effective extraction of attribute information. In contrast, although other methods detected IDC4, GAAEST, and SpaGIC incorrectly split it into two spatial domains at the boundaries, while GraphST produced overly smoothed results. spaMGCN’s ability to preserve IDC4 as a single region with well-defined boundaries highlights the advantage of its MACM module, which integrates multi-scale structural information with attribute features through stepwise fusion.

Moving to the bronchial tumor dataset, visualization of the spatial domains identified by different methods is presented in Additional file 1: Fig. S10a, while their clustering performance across six metrics is shown in Additional file 1: Fig. S10b. spaMGCN achieved the best overall performance, with an average ARI nearly 13% higher than the second-best method. Importantly, spaMGCN was the only approach that successfully grouped three discrete Bronchus regions into a single category, despite incomplete detection in two areas. This result further underscores the effectiveness of spaMGCN’s fusion of attribute and structural information.

Finally, to further explore spaMGCN’s capability in identifying spatial domains within complex tissue architectures, we analyzed the E9.5 mouse embryo dataset generated using Stereo-seq technology, which contains more than 10 distinct regions, including the brain, heart, liver, and neural crest, making spatial domain identification particularly challenging. Figure 3d presents the spatial domain delineation results from all methods, while Figs. 3e and f show the clustering metrics of spaMGCN compared to other methods. In Fig. 3d, while all methods detected cardiac tissue, spaMGCN produced significantly sharper boundaries that closely matched the ground truth annotations. Additionally, spaMGCN accurately identified the liver and connective tissues in their entirety, providing further evidence for the validity of its multi-step fusion of structural and attribute information.

Overall, these experiments demonstrate spaMGCN’s robust performance across different tissue types and dataset complexities, capable of identifying spatial domains using single-omics data while effectively integrating multi-modal information for precise spatial domain identification. The consistent improvements in boundary clarity and domain integrity highlight the advantages of its architectural design, particularly the MACM module’s ability to hierarchically combine structural and attribute features.

### spaMGCN can more accurately identify the same spatial domain that is discretely distributed within the tissue

In multicellular biological tissues, the tissue structure is generally layered and continuous [27], but there are also cell clusters that belong to the same tissue structure and perform similar functions while being discretely distributed throughout the tissue. For example, in the kidney, T cell zones play a crucial role as important aggregation areas for immune cells, responsible for monitoring and responding to pathogens and foreign substances within the system, often existing in a discretely distributed state within renal tissue. In lymph nodes, follicles are vital to the immune system, primarily composed of B cells and serving as a major source for B cell development. Follicles filter and capture antigens (such as bacteria and viruses) within the lymph nodes, ensuring that these antigens are recognized by the immune system to trigger appropriate immune responses [28, 29], and they are also discretely distributed within lymphoid tissue. Accurately identifying these discrete tissue structures is critical, as they perform significant functions.

**Fig. 4:**
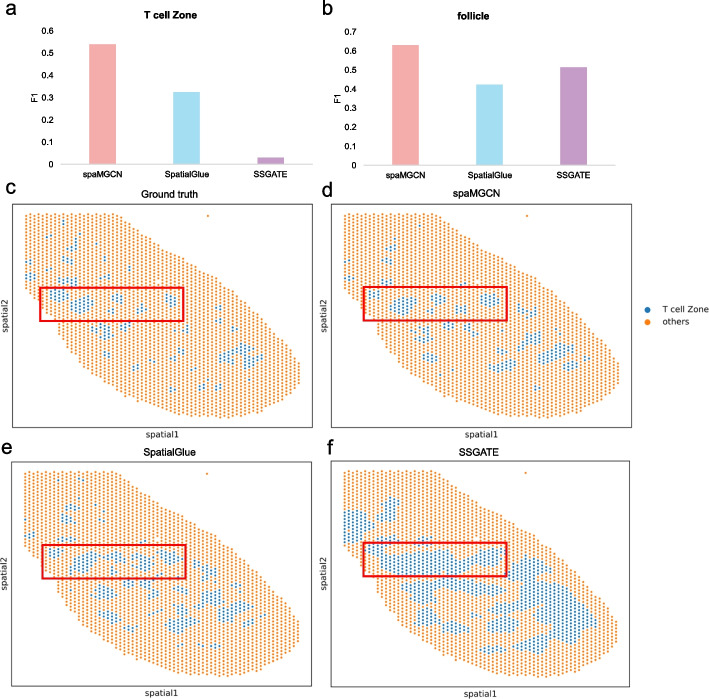
Comparison of the performance of different spatial multi-omics spatial domain identification methods for recognizing discrete spatial domains. **a** F1 scores for identifying T cell zones using different methods on the Mouse Spleen dataset. **b** F1 scores for identifying follicles using different methods on the human lymph node S3 dataset. **c** True spatial distribution of T cell zones in the Mouse Spleen dataset. **d** Spatial distribution of T cell zones identified by the spaMGCN method. **e** Spatial distribution of T cell zones identified by the SpatialGlue method. **f** Spatial distribution of T cell zones identified by the SSGATE method

To validate the effectiveness of spaMGCN in recognizing discrete tissue structures, we analyzed the Mouse Spleen and human lymph node S3 datasets. We employed various spatial domain identification methods to analyze these datasets and matched the predicted results with the true labels using the Hungarian algorithm, subsequently calculating the F1 scores for T cell zone identification on the Mouse Spleen dataset and follicle identification on the human lymph node S3 dataset, as shown in Fig. 4a and b. To visually demonstrate the performance of spaMGCN in identifying discrete spatial domains, we visualized the identification of T cell zones by spaMGCN, SpatialGlue, and SSGATE, utilizing spatial multi-omics data. Figure 4c illustrates the true distribution of T cell zones, while Figs. 4d, e, and 4f display the predictions from the three methods. Observing the red-boxed areas reveals that the predictions made by spaMGCN closely align with the true distribution. Furthermore, we analyzed the bronchus tissues in bronchial tumors. As shown in Additional file 1: Fig. S11b, only spaMGCN identified the three bronchus regions distributed in different areas as the same category. This can be attributed to the use of an autoencoder during model training to extract spot attribute information, as well as the implementation of MACM to capture multi-order neighborhood information of the spots, resulting in clear boundaries and effective classification of discrete regions under the same category. In comparison, SSTAGE, as a dual-path graph neural network model, overemphasizes the importance of spatial information while neglecting the attributes of the spots themselves, leading to unclear boundaries.

### spaMGCN can clearly separate adjacent tissue structures

**Fig. 5:**
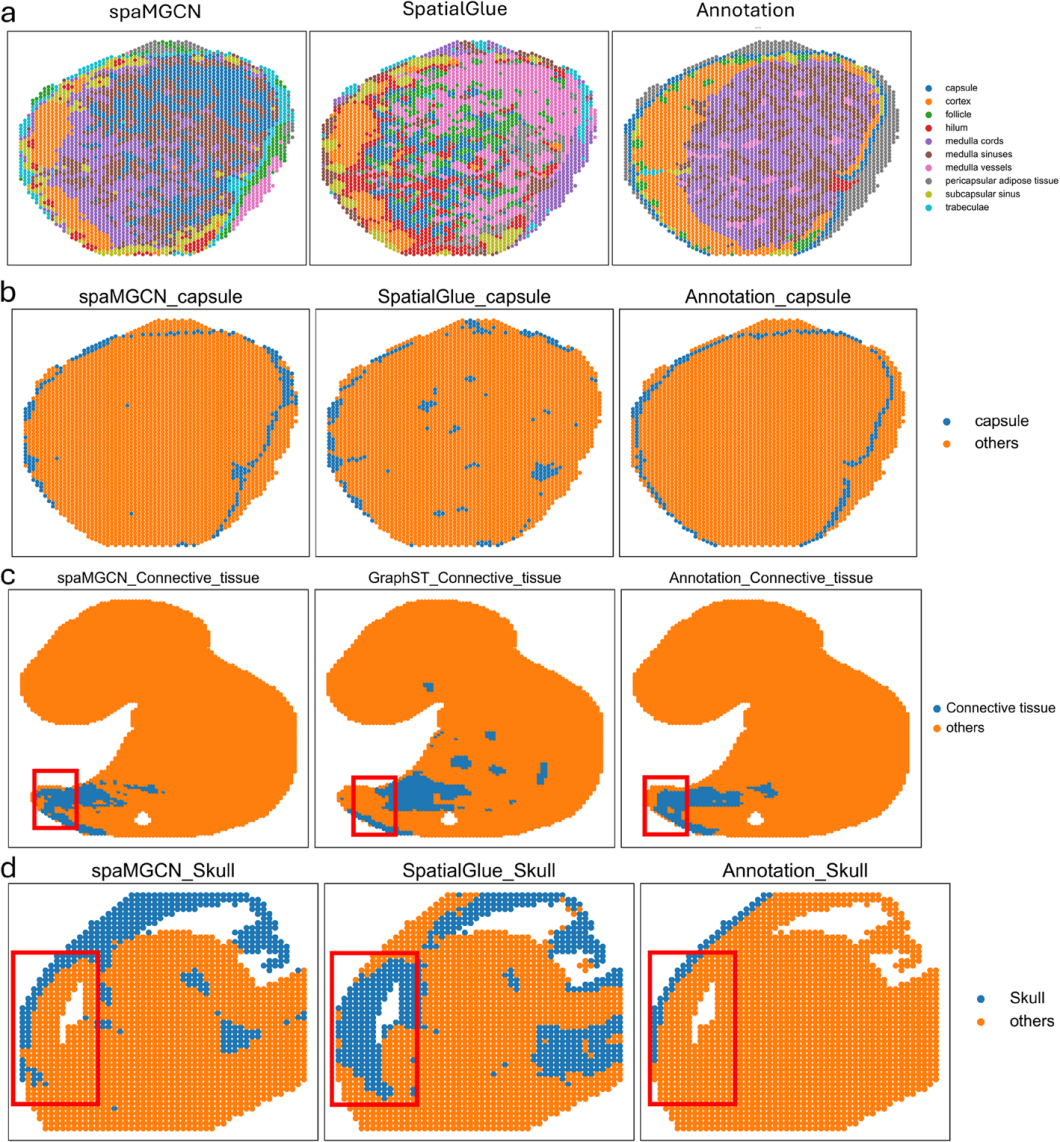
Recognition performance of different methods for the adjacent tissue structures. **a** Comparison of the spatial domains identified by spaMGCN in lymph node tissue with the actual situation. **b** Comparative analysis of capsule tissue detection by various approaches. **c** Comparative analysis of connective tissue detection by various approaches. **d** Comparative analysis of skull detection by various approaches

Lymph nodes are an essential component of the lymphatic system and play a critical role in the human immune system. They are primarily composed of the capsule, cortex, medulla, and several other small tissues. Due to the proximity of the capsule to the cortex, pericapsular adipose tissue is often categorized with these two structures during spatial domain partitioning, leading to difficulties in separating the tissue structures. To demonstrate the recognition performance of spaMGCN for adjacent tissues within lymphoid tissue, we compared its spatial domain identification with the currently top-performing method, SpatialGlue, as shown in Fig. 5. Observations from Fig. 5a reveal that spaMGCN distinctly identifies the capsule tissue without mixing it with the surrounding lymphoid adipose tissue and cortex. In contrast, the spatial domains identified by SpatialGlue group the capsule with the adjacent and structurally larger cortex and pericapsular adipose tissue. To further emphasize the recognition performance of spaMGCN for this crucial tissue structure, Fig. 5b specifically visualizes the capsule distribution identified by spaMGCN alongside the true distribution. It is evident that the capsule identified by spaMGCN closely matches the actual distribution, effectively addressing the issue of adjacent tissues being categorized as the same spatial domain. To further substantiate spaMGCN’s adjacent structure discrimination capability, we extended our analysis to the E15 mouse brain and E9.5 mouse embryo datasets (Fig. 5b,c). The forebrain, which develops into cerebral cortex and subcortical structures (basal ganglia, olfactory bulb, and hippocampus), presents particular challenges when adjacent to skull tissue. As illustrated in Fig. 5b, spaMGCN successfully separates these critical developmental structures while SpatialGlue merges them. The branchial arch, a pivotal embryonic structure that generates the six pairs of aortic arches and their associated arteries, holds fundamental importance for understanding developmental biology and the evolution of anatomical structures. As shown in Fig. 3d (E9.5 embryo), its complex adjacency with AGM, Dermomyotome, Mesenchyme, and Caity tissues creates a challenging identification scenario. Notably, Fig. 5a demonstrates that spaMGCN is the only method capable of precisely isolating this phylogenetically significant structure.

### spaMGCN can process multi-slice data in parallel and support the analysis of large datasets

In the actual analysis process, there are datasets that consist of multiple slice data. The ability to process multiple slice data in parallel will greatly simplify the analysis workflow. Therefore, we combined the adjacency matrices of three human lymph node slice datasets and constructed a multi-slice adjacency matrix using the method described in "Neighborhood graph construction" under the Methods section. Similarly, we conducted parallel analyses of the dataset containing three slice data using five-fold cross-validation with five different random seeds, and the performance results are presented in Table 1.

**Table 1:** Comparison of performance between multi-slice parallel analysis and single-slice sequential analysis

Datasets	ARI	AMI	NMI
human lymph node S1^a^	0.2627	0.4201	0.4384
human lymph node S2^a^	0.2465	0.3272	0.3486
human lymph node S3^a^	0.3272	0.4238	0.4383
human lymph node S1	0.2824	0.4025	0.4209
human lymph node S2	0.2440	0.3570	0.3779
human lymph node S3	0.2926	0.4162	0.4347

^a^indicates the parallel analysis of multiple slices

**Fig. 6:**
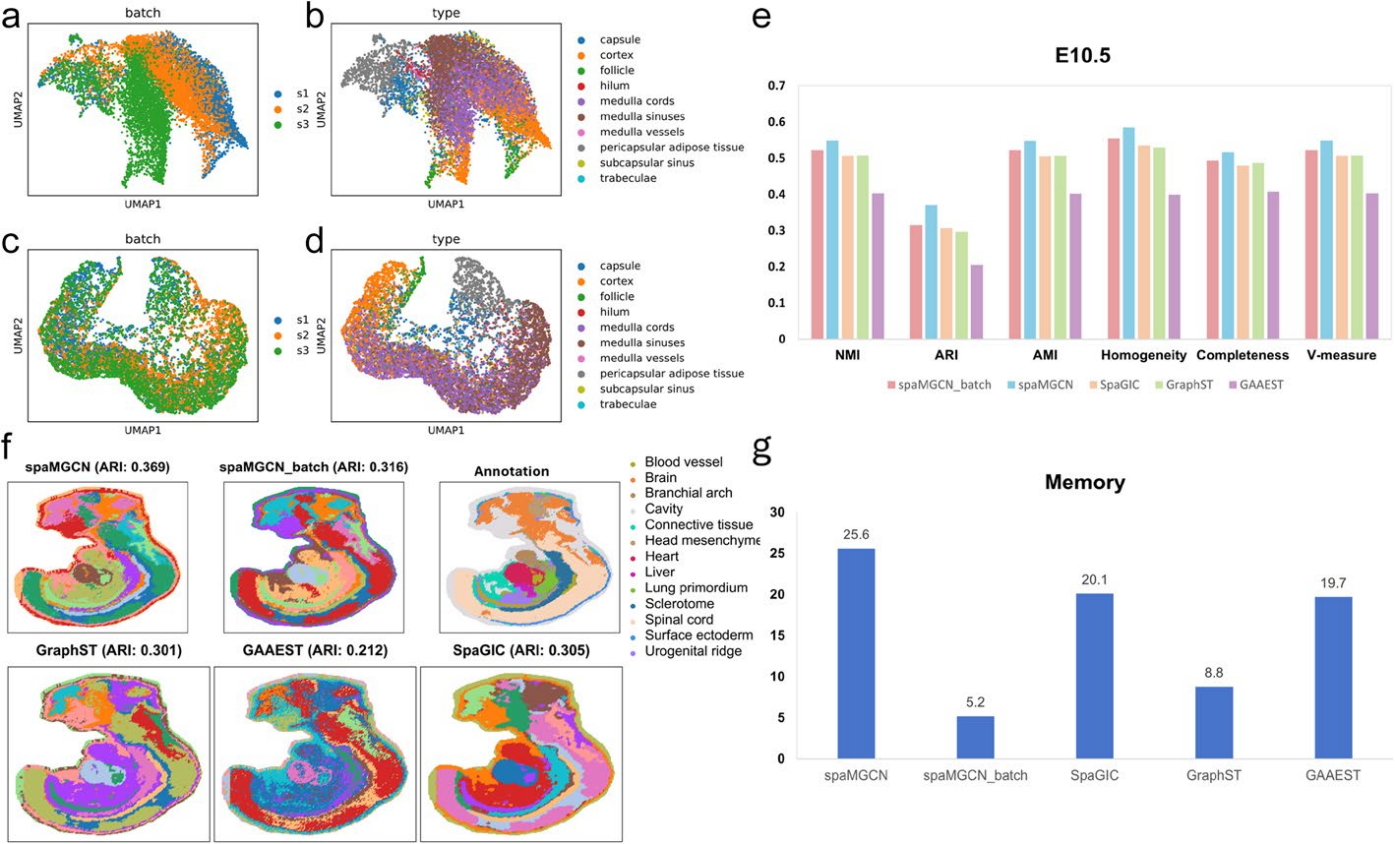
Performance analysis of spaMGCN on multi-slice and large-scale datasets. **a** Distribution of different slice data before feature reconstruction. **b** Distribution of different spatial domains before feature reconstruction. **c** Distribution of different slice data after feature reconstruction. **d** Distribution of different spatial domains after feature reconstruction. **e** Clustering performance of different methods on the E10.5 dataset. **f** Visualization of spatial domain prediction results from the spaMGCN method compared to other methods on the E10.5 dataset. **e** Memory requirements of different methods for processing the E10.5 dataset

Observation of Table 1 indicates that the performance during the parallel analysis of multiple slices is comparable to that of individual analyses, demonstrating that spaMGCN can perform parallel analyses on multi-slice datasets while maintaining equivalent spatial domain identification performance. Additionally, during the experiment, we utilized UMAP to visualize the data distribution of the multi-slice dataset before and after reconstruction, as shown in Fig. 6a–d.

Observation of Fig. 6a reveals that the data distribution of Slice 3 is significantly deviating from that of Slices 1 and 2, which is attributable to the presence of batch effects. Figure 6c illustrates the data distribution of the three slices after reconstruction with spaMGCN, showing that the data from all three slices is mixed together, successfully removing the batch effects. Furthermore, a comparison of Fig. 6b and d clearly indicates that the spots residing in the same spatial domain after data reconstruction are clustering together, providing evidence that spaMGCN can interpolate transcriptomic data, resulting in a reconstructed dataset that better reflects the true biological distribution. These findings further demonstrate that spaMGCN exhibits a competitive advantage in reducing data noise.

To evaluate scalability, we tested spaMGCN on the E10.5 mouse embryo dataset (18,408 spots × 25,201 genes). While achieving superior performance (Fig. 6e–g), the baseline implementation required 25.6 GB memory. To enable arbitrary-scale analysis, we developed spaMGCN_batch with batch training (batch_size = 5000), reducing memory usage to 5.2 GB. Although showing marginally reduced clustering metrics (Fig. 6e), spaMGCN_batch maintained precise identification of critical structures (heart, liver, notochord) while achieving 80% memory reduction. Overall, spaMGCN can handle datasets of arbitrary scale through batch processing.

### spaMGCN is capable of identifying spatial domains in single-cell resolution datasets

**Fig. 7:**
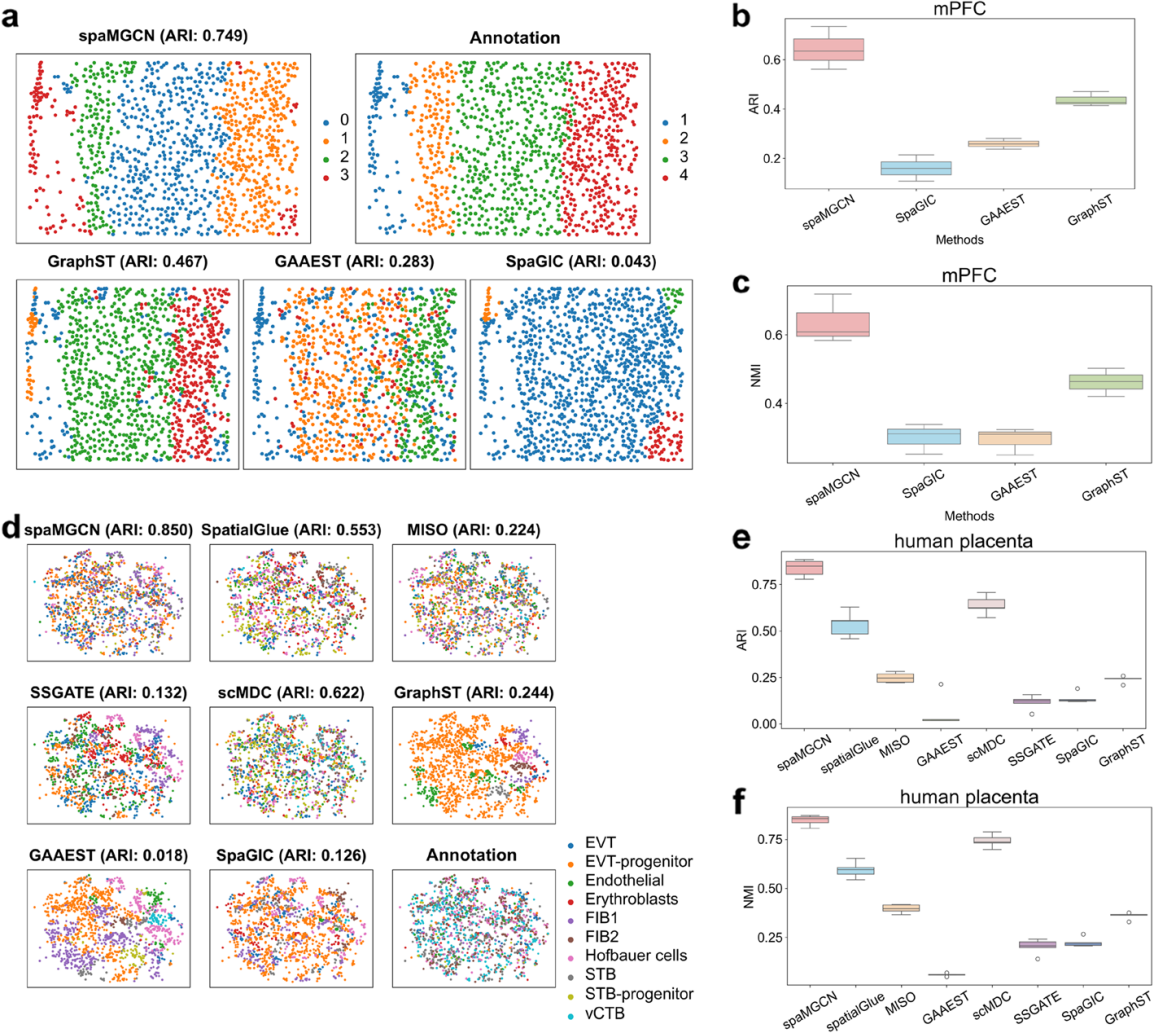
Performance comparison of different methods on single-cell resolution datasets. **a** Visualizing the spatial domain identification results of different methods on the BZ5 dataset. **b** ARI metrics of different methods on the mPFC datasets. **c** NMI metrics of different methods on the mPFC datasets. **d** Visualizing the spatial domain identification results of different methods on the human placenta dataset. **e** ARI metrics of different methods on the human placenta dataset. **f** ARI metrics of different methods on the human placenta dataset

To validate the applicability of spaMGCN to high-resolution spatial omics data, we first conducted experiments on three mouse medial prefrontal cortex (mPFC) datasets (BZ5, BZ9, and BZ14) generated by STARmap. Although these datasets had relatively low gene coverage (only 337 genes), spaMGCN achieved a median ARI of 0.749 on the BZ5 dataset, outperforming the other three methods across all six clustering metrics. Figure 7b,c and Additional file 1: Fig. S12 present boxplots comparing the average performance of spaMGCN and other methods across the three datasets. As shown in Fig. 7a, spaMGCN successfully separated the boundary between Domain 1 and Domain 2 in the BZ5 dataset, with the identified spatial domains showing high consistency with the ground truth annotations, whereas GraphST, GAAEST, and SpaGIC incorrectly merged these two domains.

To further demonstrate spaMGCN’s adaptability to single-cell resolution spatial transcriptomic data from different sequencing technologies, we applied it to three adjacent sections of the mouse hypothalamic preoptic area obtained via MERFISH. These sections contained 155 genes and were annotated with eight distinct structures: the third ventricle (V3), bed nucleus of the stria terminalis (BST), fornix columns (fx), medial preoptic area (MPA), medial preoptic nucleus (MPN), periventricular hypothalamic nucleus (PV), paraventricular hypothalamic nucleus (PVH), and paraventricular thalamic nucleus (PVT). spaMGCN achieved the best performance across all three datasets, as shown in Additional file 1: Fig. S13. On the merfish-0.14 dataset, spaMGCN attained a median ARI of 0.507, surpassing the second-best method, SpaGIC, which had an ARI of 0.309. Moreover, the spatial domains identified by spaMGCN were consistent with the ground truth annotations and exhibited symmetrical structures (Additional file 1: Fig. S14).

Finally, to evaluate spaMGCN’s capability in identifying cell types rather than relatively clustered tissue structures, we tested it on three additional single-cell resolution spatial transcriptomic datasets (Human Melanoma, humancortex, and hippocampus) generated by 10 × Genomics combined with Slide-seq technology. For these datasets, the annotations were based on individual cells rather than tissue structures. Different cell types are more dispersed compared to discrete tissues, posing significant challenges for methods that rely on spatial location information and graph neural networks for spatial domain identification. Through its multi-step fusion approach that integrates multi-level structural information with attribute information, spaMGCN achieved a median ARI of 0.7774 on the Human Melanoma dataset, which was 0.22 higher than that of the second-best method, GAAEST (ARI = 0.5464). Additional file 1: Fig. S15a, S15c, and S15e display the spatial distributions of cell types identified by different methods across the Human Melanoma, humancortex, and hippocampus datasets. Additional file 1: Fig. S15b, S15d, and S15f present UMAP visualizations of spaMGCN’s results for the three datasets, demonstrating that spaMGCN effectively separated different cell types in the feature space, with cells of the same type clustering together. These experiments on cell type-annotated spatial transcriptomic datasets further confirmed spaMGCN’s effectiveness in identifying discrete spatial domains.

Additionally, we analyzed high-resolution spatial multi-omics data from human placenta based on recent research by Johain et al. Fig. 7d compares the spatial domain identification results of different methods with the ground truth annotations. Methods that rely solely on spatial transcriptomic data (GraphST, GAAEST, and SpaGIC) incorrectly grouped large numbers of non-homologous cells into the same category due to their use of single-omics data and graph structures, resulting in overly clustered cell type identification. Although scMDC and MISO integrated two omics data types, their failure to utilize spatial information led to suboptimal performance. Figure 7e, f and Additional file 1: Fig. S16a-d present the clustering performance of different methods. Most methods that employed multiple omics data types outperformed single-omics methods on the high-resolution spatial multi-omics dataset. Our proposed spatial multi-omics clustering method, spaMGCN, achieved the best performance, which can be attributed to its ability to leverage MACM for extracting multi-scale structural information to mitigate the misclassification of adjacent cells that do not belong to the same type. The incorporation of attribute information enables spaMGCN to emphasize the importance of cell-specific characteristics, making it suitable for handling such highly discrete distributions. SpatialGlue implicitly utilizes attribute information through feature graphs; however, the construction of feature graphs is susceptible to various noise effects, potentially leading to the identification of neighboring cells that are not of the same type. Consequently, cells may lose their unique attribute information during graph convolution. To visually compare the distributions of different cell types in the feature spaces of various methods, we performed UMAP visualization for several multi-omics methods (Additional file 1: Fig. S16e-i). In the feature space extracted by spaMGCN, different cell types were well separated. Finally, to further demonstrate spaMGCN’s clustering capability on high-resolution spatial multi-omics data, we visualized the clustering results of different methods (Additional file 1: Fig. S17).

### Identification of specific biomarkers in different spatial domains

**Fig. 8:**
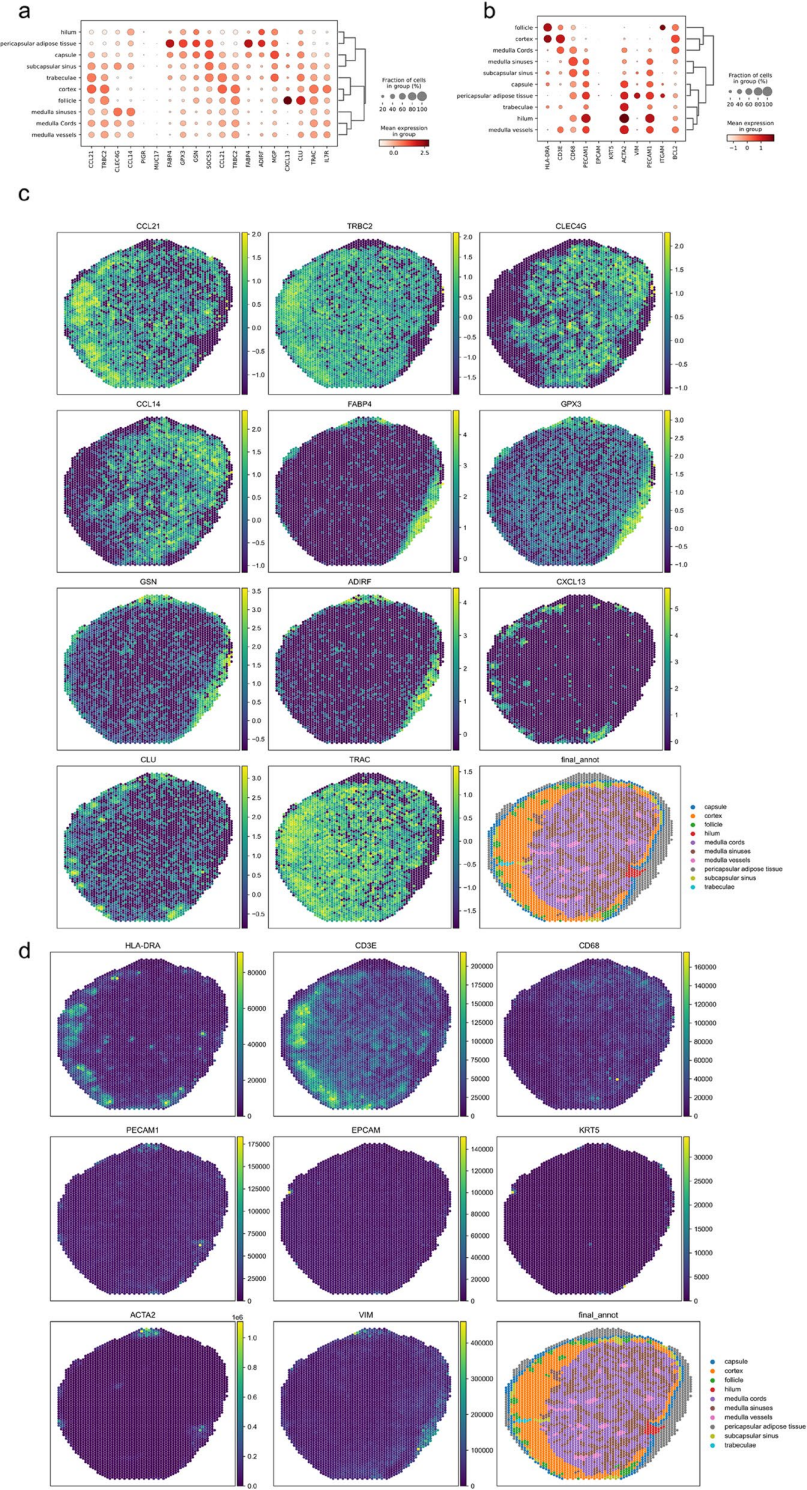
Differential analysis of spatial domains defined by spaMGCN. **a** Bubble plot of differentially expressed genes. **b** Bubble plot of differentially expressed proteins. **c** Spatial visualization of highly correlated differentially expressed genes. **d** Spatial visualization of highly correlated differentially expressed proteins

Marker genes can be used to annotate specific cell types, and by studying their expression patterns, we can elucidate cellular heterogeneity and potential gene regulatory mechanisms [30]. There is also a spatial pattern of specific gene markers that distinguishes different spatial domains. To further validate the accuracy of the spatial domain delineation obtained by spaMGCN, we employed the non-parametric Wilcoxon rank-sum test to select the top three differential features (genes, surface proteins, chromatin accessible regions) for each predicted spatial domain, verifying their association with true tissue structures through previous research publications. Additional file 1: Fig. S18-S19 display the top three differential genes and surface proteins identified through the analysis of the spatial domains defined by spaMGCN. Additionally, we plotted bubble charts of the differential genes and surface proteins, as shown in Fig. 8a and b. Some of the predicted domain-specific features exhibited significant differences across different spatial domains, thereby identifying specific spatial domains to some extent, which may represent potential spatial domain-specific genes and surface proteins.

We also visualized the spatial distribution of several differential genes that are strongly correlated with the true spatial domains. For example, CCL21, TRBC2, and TRAC clearly delineate the Cortex region in Fig. 8c. Previous studies have shown that CCL21 is primarily expressed in lymphatic endothelial cells [31], which aligns with the annotated regions. The spatial distribution of CLEC4G and CCL14 corresponds with the medulla region; the CLEC4G [32] gene encodes a member of the glycan-binding receptors and the C-type lectin family, both of which play roles in immune responses, thus aligning closely with the function of the medulla in providing lymphocytes. Conversely, the spatial distribution patterns of FABP4, GPX3, GSN, and ADIRF match those of pericapsular adipose tissue, where FABP4 [33] is primarily expressed in adipocyte cells. ADIRF plays a role in adipocyte development [32], facilitating adipogenic differentiation during the early stages of pre-adipocyte differentiation and stimulating major adipogenic factors, which is consistent with the fact that pericapsular adipose tissue is predominantly composed of adipocytes. The distribution of CXCL13 [34] aligns with the follicle region; it serves as a marker of T cell exhaustion and is utilized for the homing and localization of lymphocytes within follicles in secondary lymphoid tissues [35].

Through the analysis of the spatial domains delineated by spaMGCN, we identified several marker genes that are strongly associated with the functions of these spatial domains or have been previously confirmed. This further substantiates the efficacy of spaMGCN in recognizing spatial domains that align with biological realities. Additionally, these domain-specific genes can assist biologists in elucidating the cellular composition of different tissue structures, providing robust support for further understanding of tissue heterogeneity.

Simultaneously, we conducted differential analysis of surface protein data in the human lymph node dataset. Figure 8b presents the spatial differential proteins identified through spatial domain analysis with spaMGCN, revealing that some surface proteins exhibit strong associations with specific regions. We further visualized these highly correlated spatial differential proteins, as shown in Fig. 8d. HLA-DRA [36] is predominantly expressed in B lymphocytes, which aligns with the fact that the Cortex region is primarily composed of B cells, indirectly reflecting that the spatial domain delineation by spaMGCN is biologically grounded. Additionally, in Fig. 8d, the spatial distribution of HLA-DRA and CD3E proteins corresponds with the Cortex region and more effectively identifies the Cortex compared to the CCL21 gene. This underscores the necessity of integrating multiple omics data for spatial domain delineation and explains why the spatial domain boundaries derived from diverse omics data are more distinct and consistent with biological realities. During the differential analysis, we discovered that the distribution and associated functions of some spatial differential features found within the spatial domains delineated by spaMGCN exhibit strong correlations with the true spatial domains, further substantiating the biological relevance of the spatial domains defined by spaMGCN in alignment with biological facts. Moreover, some genes and surface proteins with spatial expression patterns, although lacking prior research linking them to specific tissue structures, display spatial distribution patterns that closely resemble true biological tissues. This suggests the potential for these to serve as spatial domain marker features, indicating that the spaMGCN model possesses significant potential value and application prospects for the identification of spatial domain-specific characteristics, which may provide direction for biologists.

We further conducted differential analysis of spatial domains identified by spaMGCN in the E15 mouse brain dataset (15-day embryonic development), focusing specifically on examining correlations and divergences between multi-omics features within each domain to validate the necessity of integrated spatial multi-omics analysis. Additional file 1: Fig. S20-S21 present domain-specific differential genes and chromatin accessible regions respectively, while Additional file 1: Fig. S22 provides integrated visualization for cross-omics comparison.

In Domain 0, the chromatin region chr8:104,022,942–104023442 (within the Gm32531 non-coding RNA) [37] showed an open state in Cartilage but was closed in adjacent Muscle tissue, clearly distinguishing these regions. In contrast, differential genes in Domain 0 covered both tissues without clear separation. Domain 2 displayed limited spatial specificity in differential chromatin regions, though genes Tenm3 and Lef1 specifically marked parts of forebrain. In Domain 4, both the chromatin regions (chr17:56,509,657–56510157 and chr5:116,895,255–116,895,755) and genes (Nfib and Igfbpl1) co-localized at the forebrain-skull boundary. Similarly in Domain 6, the chromatin region chr7:29,123,693–29,124,193 (overlapping with enhancer ENSMUSR7_C4W2S) [37] and gene Ttn both specifically marked Muscle regions. These observations demonstrate how integrating consistent multi-omics signals leads to clearer spatial domain boundaries.

### Parameter analysis and ablation study

**Fig. 9:**
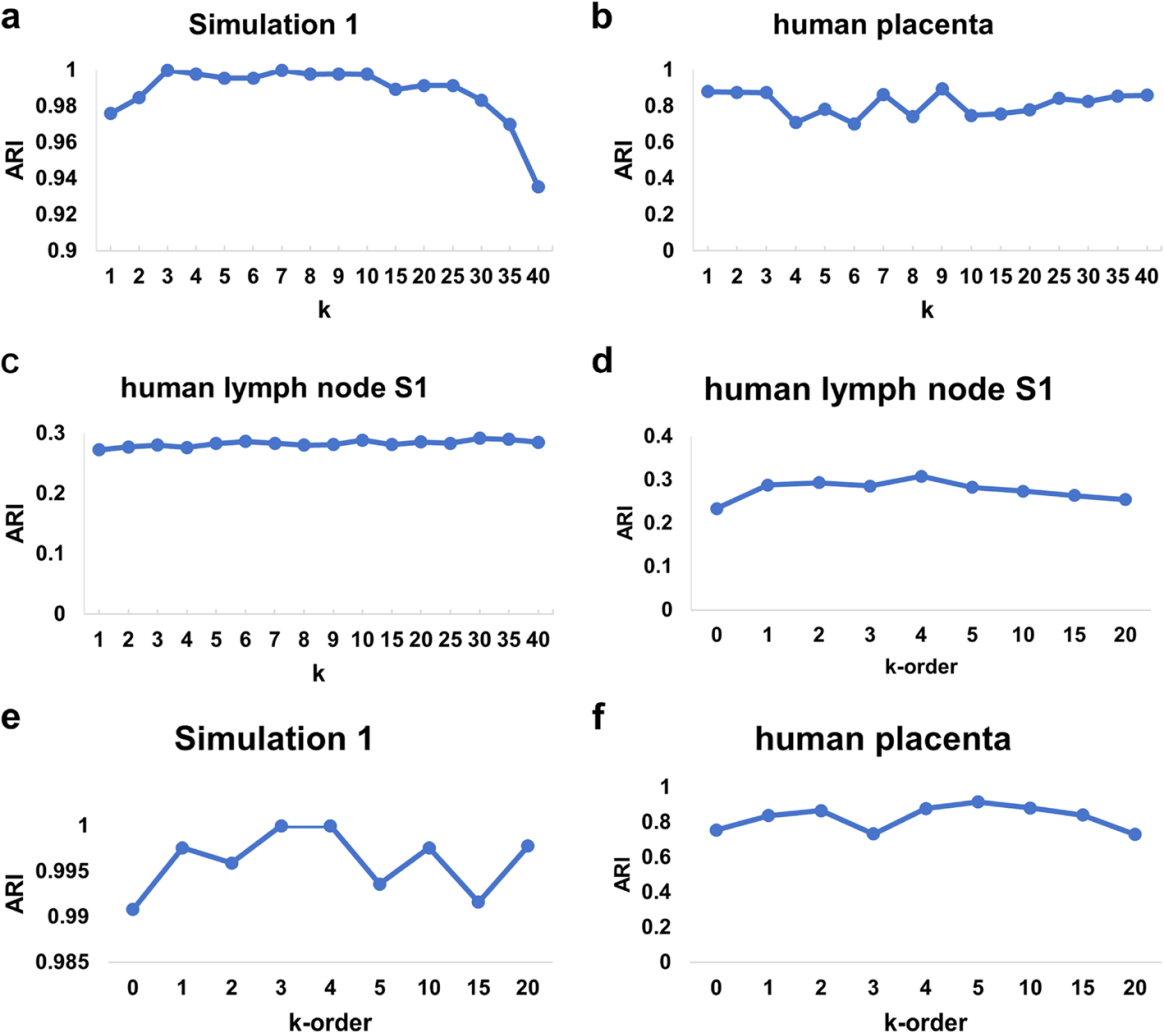
The performance of spaMGCN under different K values and K-order. **a** ARI variations of spaMGCN with different *k* values on the simulated dataset. **b** ARI variations of spaMGCN with different *k* values on the human placenta dataset. **c** ARI variations of spaMGCN with different *k* values on the human lymph node dataset S1. **d** ARI variations of spaMGCN with different *k* orders on the human lymph node dataset S1. **e** ARI variations of spaMGCN with different *k* orders on the simulated dataset. **f** ARI variations of spaMGCN with different *k* orders on the human placenta dataset

We first evaluated spaMGCN’s performance with different *k*-values for adjacency graph construction on the S1, simulated, and human placental datasets. Figure 9a shows ARI variation on the simulated dataset—optimal performance was achieved at *k* = 3, followed by a plateau and gradual decline when *k* > 25. In the human placental dataset (Fig. 9b), ARI remained consistent at *k* = 1–3 but deteriorated thereafter, as cell-type annotations in this discretely distributed tissue make adjacent cells often heterogeneous. For lymph node S1 (Fig. 9c), performance remained stable across *k*-values, likely due to inherent dataset complexity where all methods achieved ARI < 0.4.

We then assessed the *k*-order parameter in MACM, which controls adaptive integration of *k*th order neighborhood information. Figures 9d, e demonstrate performance across datasets: optimal ARI occurred at *k*-order = 4 for S1 and simulated datasets, while the placental dataset peaked at *k*-order = 5 (with *k*-order = 4 being suboptimal). Thus, we set *k*-order = 4 as default.

The sigma parameter, balancing attribute vs. structural information weight, was evaluated in Additional file 1: Fig. S23. The simulated dataset (with spatially clustered spots) favored structural information, while the placental dataset (discrete single-cell resolution) required stronger attribute information weighting.

Deep learning-based methods often exhibit considerable variability across different random seeds. To assess whether spaMGCN is sensitive to random seed selection, we tested spaMGCN on four datasets using five consecutive random seeds from 2020 to 2024. Additional file 1: Fig. S24a–d respectively show spaMGCN’s clustering performance on a simulated dataset, a human lymph node dataset, a human placenta multi-omics dataset, and a human breast cancer spatial transcriptomics dataset. On the simulated dataset, human lymph node dataset, and human breast cancer dataset, spaMGCN is relatively insensitive to the choice of random seed, with the maximum difference between seeds not exceeding 0.08. However, on the human placenta single-cell resolution spatial multi-omics dataset, spaMGCN is more sensitive, with a maximum difference of 0.11 between the highest and lowest values. Together with the line charts and the earlier boxplots, these results indicate that spaMGCN is generally not very sensitive to random seed selection across most datasets.

To validate module contributions, we tested five spaMGCN variants:

- Without MACM (w/o MACM): removed multi-scale adaptive convolution.
- Without MAF (w/o MAF): disabled multi-step attribute fusion.
- Without AI (w/o AI): eliminated attribute information.
- Without NCE (w/o NCE): removed InfoNCE contrastive loss.
- Without BCE (w/o BCE): omitted binary cross-entropy loss.

**Fig. 10:**
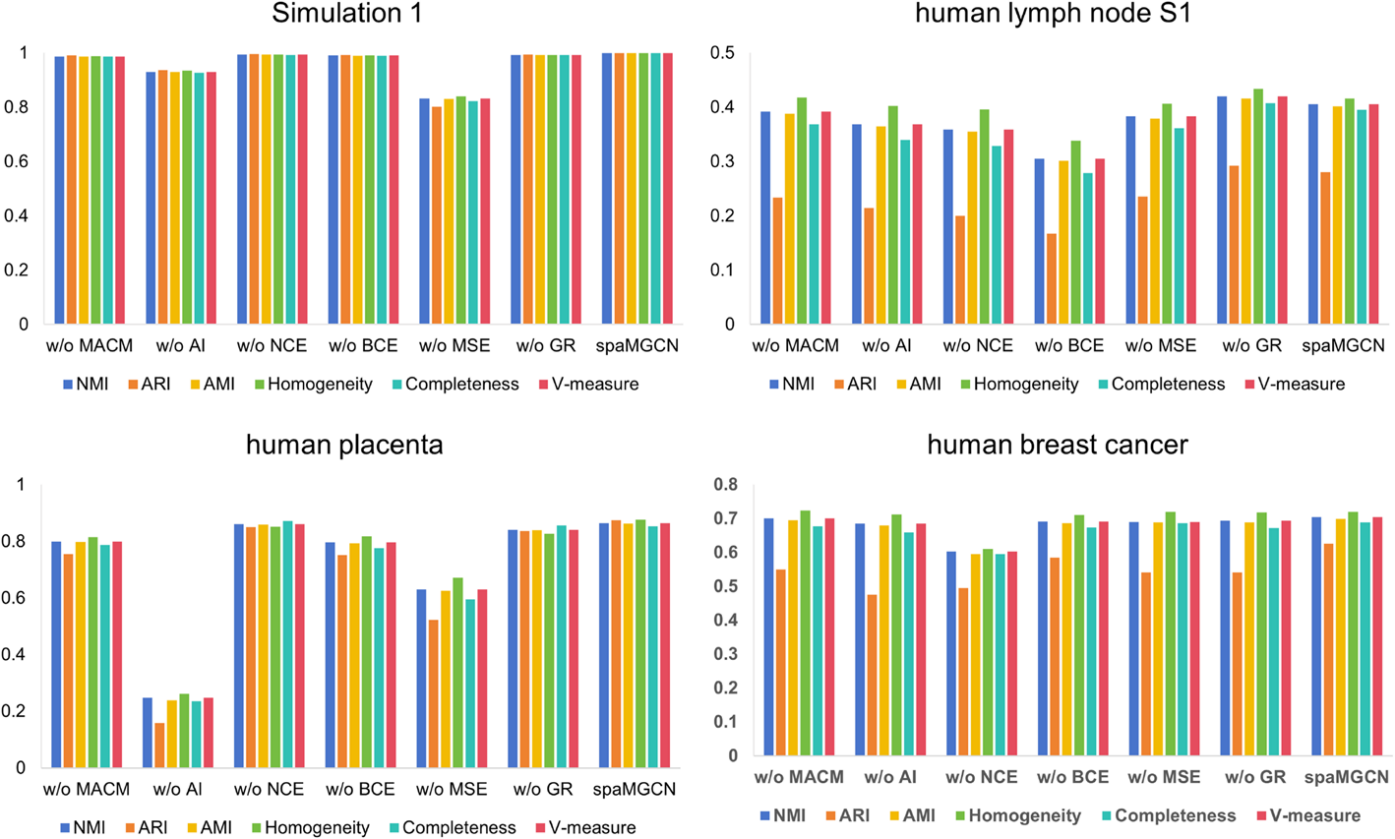
Ablation experiments conducted on four datasets

We conducted comprehensive ablation experiments across four representative dataset types—simulated data, spot-level spatial multi-omics (human lymph node S1), single-cell resolution multi-omics (human embryo), and spatial transcriptomics (human breast cancer)—with results in Fig. 10 conclusively demonstrating each component’s necessity for optimal performance, particularly highlighting the critical contributions of the MACM module and multi-step attribute fusion mechanism to spaMGCN’s robust performance across different data modalities and resolutions. The MACM module is an important component of spaMGCN designed to capture multi-scale structural information. We believe it outperforms traditional GAT or GCN modules when handling datasets with irregular spatial distributions because it can weight neighborhood information at different levels. To further demonstrate the effectiveness of the MACM module, we first performed *t*-tests on the results from the simulated dataset to verify that the impact of the MACM module is significant, as shown in Additional file 1: Fig. S25. The human lymph node dataset matches our expectations, containing both spatially clustered and spatially dispersed tissues. We conducted *t*-tests on the ablation results of the MACM module across three slices of this dataset, as shown in Additional file 1: Fig. S26–S28. Except for the Homogeneity metric on the S1 slice, all other metrics significantly outperform the spaMGCN variant without the MACM module.

## Discussion

Accurate identification of spatial domains is fundamental to understanding tissue composition and heterogeneity. To this end, we propose a deep learning method for spatial domain delineation that utilizes spatial transcriptomics and spatial epigenomics data—spaMGCN. This method consists of a set of parallel feature extraction modules, each designed to extract multi-source fusion features from different omics data. Additionally, when dealing with single omics data, spaMGCN achieves analysis by removing one of the feature extraction modules. During the training process, spaMGCN employs a graph-structured binary cross-entropy loss, enhancing the mutual information between adjacent points (spots) and ensuring that physically proximate points are more compact in the feature space, thereby guaranteeing the accuracy of the model in recognizing contiguous spatial domains. In addition, to avoid the over-smoothing phenomenon that may arise from traditional graph convolutional networks, spaMGCN incorporates a MACM module to capture multi-order neighbor information from different points, thoroughly considering the contributions of various neighbors to the central node. This ensures that the recognized spatial domain boundaries are clear, preventing all neighbors around a node from being grouped into a single spatial domain. Concurrently, we utilize autoencoders to capture attribute information from different points and facilitate multi-step integration through several skip connections with the multi-scale structural information obtained from the MACM module. This design mitigates the risk of overemphasizing structural information, ensuring clear spatial domain boundaries and avoiding the misidentification of subtle spatial domains and the partitioning of functionally similar but spatially discrete areas into multiple spatial domains. Ultimately, spaMGCN consolidates the fusion features derived from various omics data, achieving spatial domain delineation that aligns more closely with biological realities.

Through experiments conducted on multiple sequencing platforms, we demonstrated the robust performance of spaMGCN in spatial domain identification. In the human lymph node datasets S1 and S3, the clustering accuracy of spaMGCN improved by 5 and 6%, respectively, further validating its superior performance. Particularly in dissecting the organizational structure of human lymph nodes, spaMGCN effectively distinguished between the capsule and the adjacent cortex as well as the pericapsular adipose tissue, an achievement that is challenging for other spatial domain identification methods. Additionally, spaMGCN was able to clearly identify the discretely distributed T cell zones within kidney tissue and the discrete follicles within lymph node tissue. For these two spatial domains, spaMGCN outperformed the second-best method, achieving F1 scores that were higher by 21.6 and 11.6%, respectively, thus further demonstrating its exceptional capability in recognizing discrete spatial domains. These outstanding results can be attributed to spaMGCN’s comprehensive integration of attribute information through multi-step fusion, taking into account various omics data. Moreover, we enhanced the applicability of spaMGCN by allowing it to process single omics data through the removal of one feature extraction module. When analyzing the human breast cancer dataset, spaMGCN successfully identified the IDC_5 tumor region and the associated edge region Tumor_edge_4, while other methods mixed these two domains. In the E9.5 mouse embryo dataset, spaMGCN effectively separated connective tissue from other adjacent tissues. During the analysis of the bronchial tumor dataset, only spaMGCN was able to recognize the three discrete bronchus regions, even though two of the areas were not complete.

To assess the scalability of spaMGCN, we conducted experiments on a human lymph node dataset containing three slices. The results showed that spaMGCN achieved performance comparable to single-slice analyses while effectively eliminating batch effects in multi-slice transcriptomic data. Additionally, we analyzed the memory requirements of different methods when faced with a large-scale dataset, specifically the E10.5 mouse embryo dataset (18,408 spots × 25,201 genes). spaMGCN consumed the most memory at 25.6 GB, but our variant spaMGCN_batch was able to process this large-scale dataset with only 5.2 GB of memory when the batch_size was set to 5000, successfully identifying important tissues such as the heart and liver, despite a slight decrease in clustering metrics. The batch processing mechanism of spaMGCN provides feasibility for handling even larger datasets in the future. Furthermore, we applied spaMGCN to the latest human placenta spatial multi-omics dataset with single-cell resolution, which includes transcriptomics and chromatin accessibility data, as well as a series of single-cell resolution spatial transcriptomic datasets obtained from Slide-tag technology. spaMGCN achieved the best performance across all of these datasets, further confirming its versatility and capability to capture fine-grained features.

Lastly, we performed differential analysis on the spatial domains delineated by spaMGCN, validating the top-ranked differential features and providing a biological basis for the spatial domain delineation, which further confirmed the accuracy of spaMGCN. For some unverified differential features, we utilized the scanpy package for spatial visualization and discovered significant differences in these features across various spatial domains, suggesting their potential as domain-specific markers and offering valuable insights for biologists. At the same time, we visualized the distribution of different omic differential features within the same domain in the E15 dataset, further demonstrating the necessity of integrating spatial multi-omics data for spatial domain identification. Across all these datasets, spaMGCN consistently delivered superior performance, demonstrating both its exceptional versatility in handling diverse data types and its capability to capture fine-grained biological features.

Currently, sequencing technologies for spatial multi-omics data are becoming increasingly mature, and spaMGCN is expected to be compatible with diverse types and platforms of spatial multi-omics data in the future. Moreover, integrating additional omics data will provide richer information, facilitating the interpretation of tissue heterogeneity from multiple perspectives and enabling more accurate spatial domain delineation. Although we applied our method to the three-omics dataset generated by SpatialGlue, the performance was unsatisfactory. We speculate that the poor performance may be due to our use of the simplest direct concatenation method for feature fusion, while significant differences exist between omics modalities. This makes it difficult for the approach to fully mine and integrate information when dealing with tri-modal data. To address this issue, in the future we will focus on modality alignment and data fusion to optimize the design of spaMGCN, enhancing its ability to adapt to differences across multiple modalities and thereby more effectively process spatial multi-omics datasets that contain diverse and significantly heterogeneous data types. Additionally, many current spatial transcriptomic datasets include hematoxylin and eosin (H&E)-stained image data, yet there remains a lack of reliable methods to effectively integrate such image data for spatial domain identification. In future work, we will focus on advancing these research directions to further improve the scalability of spaMGCN.

## Conclusion

In summary, to our knowledge, spaMGCN is the first spatial domain partitioning method that focuses on recognizing discrete distribution spots within the same spatial domain. It provides a novel approach for identifying complex spatial structures and spatial domains with discrete distributions. We conducted experiments using spatial multi-omics data generated across multiple platforms and a series of spatial transcriptomics datasets, confirming the advantages of spaMGCN and highlighting its potential for comprehensive utilization of spatial multi-omics data.

## Methods

### Data preprocessing

Data preprocessing operations are a crucial step in data analysis, providing high-quality data for the training of the spaMGCN model. The input to the spaMGCN model consists of gene expression matrices, other omics data matrices, and spatial location information. We adopted the same data processing approach as SpatialGlue [11]. For gene expression data, we first filtered out genes expressed in fewer than 10 spots. We then performed log transformation and normalization on the filtered gene expression counts using the SCANPY [38] package. Additionally, to focus more on the genes that contribute significantly to cellular variation, we selected the top 3000 highly variable genes (HVGs) to be used as input for principal component analysis (PCA) [39], with the PCA dimensions set to match the feature dimensions of the proteomics data. For proteomics data, we applied centered log-ratio normalization to the raw protein expression counts. We then executed PCA on the normalized data and used the first *n* − 1 principal components as input to the encoder, where *n* represents the original feature dimension of the proteomics data. For the human placental dataset, we utilized the gene scores generated from ATAC-seq data provided by the original authors as one omics data modality, and performed the same processing for proteomics data. Regarding chromatin accessibility data, we applied latent semantic indexing to reduce the original chromatin peak count data to 200 dimensions. For spatial transcriptomics datasets with high gene capture rates, we first filtered out genes detected in fewer than 10 spots. The filtered gene expression counts were then log-transformed and normalized using the SCANPY package, followed by selection of the top 3000 highly variable genes (HVGs) for spatial domain identification. For datasets with limited gene coverage such as MERFISH and STARmap, we adopted the same processing strategy used for proteomics data.

### Neighborhood graph construction

In tissues, spatially adjacent spots typically possess similar cell types or belong to the same functional area. We utilize the *k*-nearest neighbors (KNN) algorithm to convert the spatial location information of the spots into a neighborhood graph *G* = (*V*,*E*). Here, *V* represents the set of points, *E* denotes the set of edges connecting the points, and *k* is the number of neighbors. Specifically, for a given point *ii* belonging to *V*, we calculate the Euclidean distance between this point and all other points based on spatial location information and select the *k* closest points as its neighbors. By this method, we derive the adjacency relationships between all points, and the adjacency matrix *A ϵ RN* × *N* is used to represent the adjacency relationships among all points in the neighborhood graph *G*. This is expressed as follows:

$$A_{ij}=\left\{\begin{array}{ll}1&if\;spot\;j\;\in V\;is\;the\;k-closest\;neighbor\;of\;spot\;i\\0&otherwise\end{array}\right.$$

We then normalize the adjacency matrix by adding self-loops and using the inverse square root of the degree to facilitate feature propagation within the graph neural network while also preventing gradient explosion during the training process.

When addressing the multi-slice problem,${A}_{1}$*,*${A}_{2}$*,…,*${A}_{n}$ represent the adjacency matrices of the *n* slices, and the integrated adjacency matrix *A* is constructed in the following form:

$$A=\left[\begin{array}{ccccc}{A}_{1}& 0& 0& \dots & 0\\ 0& {A}_{2}& 0& \dots & 0\\ 0& 0& {A}_{3}& \dots & 0\\ \vdots & \vdots & \vdots & \ddots & \vdots \\ 0& 0& 0& \dots & {A}_{n}\end{array}\right]$$

### Multi-scale adaptive convolution module

Inspired by the recent work of Li et al. [24], we utilized the proposed Multi-Scale Adaptive Convolution Module (MACM) to capture the structural information of the graph and to adaptively fuse multiple feature matrices that contain different-order neighborhood information, thereby obtaining more complex and comprehensive structural features. Specifically, MACM conveys multi-order neighbor information by constructing multiple high-order adjacency matrices. This approach overcomes the limitations of direct adjacency and merges multi-order neighbor information, resulting in a more complete and rich structural representation of the spots.

The implementation is as follows: Let $\text{X}\in {\text{R}}^{N\times d}$ be a feature matrix for a certain omics dataset of all spots on a tissue slice, where *N* is the number of spots and *d* is the feature dimension. The feature encoding that incorporates *k*th order neighbor information is represented as:

$${h}_{k}={A}^{k}Tanh\left(X{W}^{(k)}\right)$$

where ${h}_{k}$ represents the feature encoding that includes *k*th order neighbor information, *A* is the adjacency matrix, Tanh is the activation function, *X* is the omics feature matrix, and ${W}^{(k)}$ represents the learnable parameters for obtaining *k*th order neighbor information. Through this method, we can obtain a set of multi-scale feature encodings *H* that include different neighbor information, represented as:

$$H=\left[{h}_{1};{h}_{2};{h}_{3};\dots ;{h}_{k}\right]$$

After acquiring the multi-scale feature encoding *H*, we apply average pooling to each scale’s feature encoding and concatenate them, represented as:

$${h}_{m}=[{f}_{mean}\left({h}_{1},dim=0\right),{f}_{mean}\left({h}_{2},dim=0\right),\dots ,{f}_{mean}\left({h}_{k},dim=0\right)]$$

where ${f}_{mean}\left(.\right)$ indicates the average pooling operation applied to the feature matrix. We then utilize a MLP module to adaptively learn the importance of the feature encodings at different scales, with the importance score matrix represented as:

$$W=softmax({f}_{m}({h}_{m}))$$

Subsequently, we use the importance score matrix to fuse the multi-scale feature encodings, each containing different-order neighbor information, to obtain the final feature representation that incorporates multi-scale structural information, as follows:

$$h={h}_{1}{W}_{\left(0\right)}+{h}_{2}{W}_{\left(1\right)}+{h}_{3}{W}_{\left(3\right)}+\dots +{h}_{k}{W}_{\left(k\right)}$$

where *h* indicates the feature representation that has fused multi-scale structural information, and ${W}_{\left(k\right)}$ represents the importance of the feature matrix containing *k*th order neighborhood information. Through comprehensive evaluation of spaMGCN’s performance across multiple *k*-values, we ultimately set this parameter to 4.

### Multi-source feature fusion

The Multi-Source Feature Fusion Module, as illustrated in Fig. 1b, consists of two sets of autoencoders, a graph encoder, and a graph decoder. Both the encoder and the decoder modules of the autoencoders are composed of three fully connected layers and two LeakyReLU layers. The encoder module is designed to extract the attribute features of different spots, with the gene expression attribute feature extraction process for different spots represented simply as:

$${h}^{RNA}={f}_{e}^{RNA}\left({x}_{RNA}\right)$$

The feature extraction process for other omics data follows a similar approach; here we take protein expression as an example, represented as:

$${h}^{ADT}={f}_{e}^{ADT}\left({x}_{ADT}\right)$$

where ${f}_{e}^{RNA}\left(.\right)$ and ${f}_{e}^{ADT}\left(.\right)$ represent the encoders for different omics data, aimed at capturing various omics attribute features of different spots (ADT denotes the proteomics data modality). The decoder operates as the inverse process of the encoder, designed to reconstruct different omics data. The reconstruction process of the gene expression profile is represented as:

$${x}_{RNA}^{,}={f}_{d}^{RNA}\left({h}^{RNA}\right)$$

The reconstruction process for other omics data is:

$${x}_{ADT}^{,}={f}_{d}^{ADT}\left({h}^{ADT}\right)$$

where ${f}_{d}^{RNA}\left(.\right)$ and ${f}_{d}^{ADT}\left(.\right)$ represent different layers of the decoders for the respective omics data,${x}_{RNA}^{,}$ and ${x}_{ADT}^{,}$ denote the reconstructed data from the autoencoders for the different omics datasets. We compute the mean squared error between the reconstructed data and the original data across different omics as the reconstruction loss ${L}_{R}$ to ensure that the features extracted by the autoencoders remain consistent with the original features, represented as follows:

$${L}_{R}=\frac{1}{n}{\left|{x}_{RNA}-{x}_{RNA}{\prime}\right|}^{2}+\frac{1}{n}{\left|{x}_{ADT}-{x}_{ADT}{\prime}\right|}^{2}$$

where *n* denotes the number of spots, with the two parts representing the reconstruction loss for different omics data. The extraction of attribute information from the spots is beneficial for partitioning spatially discrete elements within the same spatial domain together.

The structural feature extraction module consists of three layers of the MACM module, which serves as an encoder for extracting structural features. During the process of extracting structural features, we perform multi-step fusion of the attribute features extracted from different layers of the autoencoder with the multi-scale structural features extracted from the MACM module. This approach ensures the sufficient integration of attribute and structural information, thereby avoiding the situation where only spatial structural information is considered while neglecting the attribute information of the spots themselves. This is represented as follows:

$${H}_{1}^{RNA}={MACM}_{1}^{RNA}({x}_{RNA},A );$$

$${H}_{i}^{RNA}={MACM}_{i}^{RNA}\left(a{H}_{i-1}^{RNA}+\left(1-a\right){h}_{i-1}^{RNA},A \right);$$

$${H}^{RNA}=a{H}_{n}^{RNA}+\left(1-a\right){h}^{RNA}$$

where *a* is a hyperparameter, *n* denotes the number of layers in the graph encoder, *A* represents the adjacency matrix constructed based on spatial location information,${H}^{RNA}$ denotes the multi-source feature fusion representation of the RNA omics data, and ${h}_{i}^{RNA}$ represents the attribute features extracted from different layers of the autoencoder. The process for extracting the multi-source fusion features ${H}^{ADT}$ from other omics data is similar. The extraction of multi-scale structural information can consider that, in reality, multicellular biological tissues are often aggregated in spatial domains, allowing the spaMGCN to maintain recognition accuracy for continuous and clustered spatial domains. Through multi-step fusion, spaMGCN comprehensively considers the attribute information and structural information of different omics data, enabling the extracted low-dimensional representation to be applied to the recognition of similar spatial domains.

Next, we utilize the graph decoder to perform node embedding reconstruction of the multi-source fusion features from different omics datasets, where the structure of the decoder is symmetric to that of the encoder, simplifying the learning process. The node embedding reconstruction is represented as follows:

$${Z}_{RNA}={G}_{D}^{RNA}\left({H}^{RNA},A \right);$$

$${Z}_{ADT}={G}_{D}^{ADT}\left({H}^{ADT},A\right)$$

where ${Z}_{RNA}$ and ${Z}_{ADT}$ denote the reconstructed omics embedding features, which contain neighbor information and related topological information.${G}_{D}^{RNA}$ and ${G}_{D}^{ADT}$ are the decoders for the omics embeddings from different datasets. We then calculate the graph feature reconstruction loss for the different omics data as follows:

$${L}_{G}=\frac{1}{n}{\left|{Z}_{RNA}-{Ax}_{RNA}\right|}^{2}+\frac{1}{n}{\left|{Z}_{ADT}-{Ax}_{ADT}\right|}^{2}$$

The graph feature reconstruction loss calculates the reconstruction loss of the omics embeddings containing neighbor information, which is crucial for a deeper understanding of the intrinsic properties of the spots and their spatial information.

### Multi-omics data fusion

To further fuse the low-dimensional representations of different omics, we first concatenate the multi-source fusion features from various omics datasets, represented as follows:

$$Z=\left[{H}^{RNA},{H}^{ADT}\right]$$

The fusion of multiple omics features allows the model to analyze the spatial domain from various perspectives. Inspired by Deep Graph Structural Infomax [25], we introduce a graph-structured constraint to facilitate the learning of node representations. We reconstruct the graph structure based on the feature representation *Z* and compute the binary cross-entropy loss as the structural reconstruction loss ${L}_{S}$, represented as follows:

$${A}^{,}=sigmoid\left(Z{Z}^{T} \right);$$

$${L}_{S}=-\frac{1}{{N}^{2}}\sum_{i=1}^{N} \sum_{j=1}^{N} \left[{A}_{ij}\cdot \text{log}\left({A}_{ij}^{,}\right)\right]+(1-{A}_{ij})\cdot \text{log}\left(1-{A}_{ij}^{,}\right)$$

where ${A}^{,}$ represents the reconstructed graph obtained from the fused features *Z*. At the same time, in order to make the feature representation *Z* of the spots more informative and discriminative, a contrastive learning loss similar to InfoNCE is computed, such that physically adjacent spots are closer to each other in the feature space, while non-adjacent spots are further apart [40]. It is expressed as follows:

$${L}_{\text{con}}=-\frac{1}{N}\sum_{i}^{N} \text{log}\frac{\sum_{j\in {N}_{i}}^{N} {\text{exp}}^{(\text{cos}({Z}_{i},{Z}_{j})/\tau )}}{\sum_{j\notin {N}_{i}}^{N} {\text{exp}}^{(\text{cos}({Z}_{i},{Z}_{j})/\tau )}}$$

where ${N}_{i}$ is the neighbor set of spot *i*,$\text{cos}({Z}_{i},{Z}_{j})$ denotes the cosine similarity between spots *i* and *j*, and *τ* represents a hyperparameter. For a given spot, its adjacent spots are defined as positive pairs, while its non-adjacent spots are defined as negative pairs. The final overall loss is defined as:

$$L={L}_{R}+{L}_{G}+{L}_{S}+{L}_{\text{con}}$$

Through the joint constraints of multiple losses, we obtain the fused feature representation *Z* that incorporates the attribute information and structural information of various omics data.

### Spatial domain identification

Finally, we perform clustering analysis on the fused features *Z* to identify the spatial domains of different tissues.

### Evaluation metrics

This study employs three widely used evaluation metrics—Adjusted Rand Index (ARI), Adjusted Mutual Information (AMI), and Normalized Mutual Information (NMI)—to assess the clustering performance of the model. The F1 score is utilized to evaluate the accuracy of discrete spatial domain identification.

The ARI evaluates clustering quality by comparing the true labels of data points with the results obtained from the clustering algorithm, calculating their similarity. It is expressed as follows:

$$ARI=\frac{2(ad-bc)}{(a+b)(b+d)+(a+c)(c+d)}$$

where *a* represents the number of point pairs that belong to the same cluster in both the true and experimental conditions, *b* denotes the number of point pairs that belong to the same cluster in the true condition but not in the experimental condition, *c* indicates the number of point pairs that do not belong to the same cluster in the true condition but do in the experimental condition, and *d* represents the number of point pairs that do not belong to the same cluster in either the true or experimental conditions.

NMI evaluates clustering quality by comparing the clustering results with the true labels using an information-theoretic measure. The calculation of NMI is based on Mutual Information (MI), which considers the information overlap between true labels and clustering results. It is expressed as:

$$NMI=\frac{2MI(U,V)}{H(U)+H(V)}$$

AMI is an adjusted version of MI, which measures the degree of mutual dependence between two random variables. It is expressed as:

$$AMI(U,V)=\frac{I(U;V)-E[I(U;V)]}{H(U)+H(V)-2E[I(U;V)]}$$

The F1 score is a commonly used metric for evaluating the performance of classification models, particularly in cases of imbalanced data. It combines the model’s precision and recall, as detailed below:

$$F1=2\times \frac{Precision\times Recall}{Precision+Recall}$$

### Benchmarking methods

In this study, we conducted benchmarking of spaMGCN against the latest methods—SpatialGlue [41], SSGATE [42], GraphST [43], GAAEST [44], SpaGIC [45], MISO [46], and scMDC [47]—using different tests with default parameters. SpatialGlue, MISO, SSGATE, GraphST, GAAEST, and SpaGIC are spatial domain identification methods, while scMDC is a single-cell multi-omics clustering method. All comparison methods were processed according to the data processing procedures specified in the original papers, adhering to the implementations described in their respective official repositories. Parameter settings were configured using the default parameters.

To optimize the performance of spaMGCN across diverse datasets, we tailored the parameter configurations as follows: For the construction of the *k*-nearest neighbor (KNN) graph, we set *k* = 3 while the MACM module utilized *k* = 4. The sigma was adjusted according to dataset characteristics: sigma = 0.7 for the human lymph node, mouse spleen, and mouse thymus datasets; sigma = 0.5 for the simulated dataset, mouse brain E15, and mouse medial prefrontal cortex (mPFC) sections; sigma = 0.3 for the human placenta, human breast cancer, and human bronchial tumor datasets; and sigma = 0.2 for the mouse embryo datasets (E9.5 and E10.5). For the MERFISH series, human melanoma, human cortex, and hippocampus datasets, sigma was set to 0.4.

## Supplementary Information

Additional file 1: Fig. S1-S28.

Additional file 2: Table S1-S4.

## Data Availability

We obtained six multi-omics datasets from the authors of SpatialGlue [11], SpaMosaic [48] (https://github.com/JinmiaoChenLab) and spaVAE [49]. These datasets were generated using three spatial multi-omics sequencing technologies: 10x Genomics Visium, MISAR-seq, Stereo-CITE-seq, and SPOTS, which can simultaneously measure protein markers, transcriptomes, and spatial location information. Specifically, the Mouse Thymus dataset was obtained using Stereo-CITE-seq technology, the Mouse Spleen dataset was measured using SPOTS technology, and the human lymph node dataset consists of data from three tissue sections, all measured using 10x Genomics Visium technology. All the above data can be obtained at https://zenodo.org/records/10362607 [50] and 10.5281/zenodo.7480069 [51]. Additionally, we acquired a human placenta multi-omics dataset with single-cell resolution, referred to as human_placenta, from Johain R et al. [52] (https://singlecell.broadinstitute.org/single_cell/study/SCP2601). This dataset provides gene expression data, chromatin accessibility data, and spatial location information of the cells. The spatial transcriptomics datasets were obtained from publicly available sources: the mouse embryo datasets 8(E9.5 and E10.5) were downloaded from the MOSTA database (https://db.cngb.org/stomics/mosta/download/) [53], the 10x Visium human breast cancer datasets were obtained from the 10x Genomics official portal (https://www.10xgenomics.com/datasets/human-breast-cancer-block-a-section-1-1-standard-1-1-0), the Bronchiolar dataset was retrieved from Zenodo (https://zenodo.org/records/8185216) [54], the three mouse medial prefrontal cortex (mPFC) sections and MERFISH data were acquired from the STAGUE GitHub repository (https://github.com/deepomicslab/STAGUE )[55], and the Human Melanoma, humancortex, and hippocampus datasets were sourced from the Broad Institute Single Cell Portal (https://singlecell.broadinstitute.org/single_cell/study/SCP2162). An open-source software implementation of spaMGCN is publicly available under the MIT license on GitHub: https://github.com/hongfeiZhang-source/spaMGCN [56] and Zenodo: 10.5281/zenodo.15112750 [57]. For more detailed data information, please refer to Additional file 2: Table S4.
