# Supplementary material for: spaMGCN: a graph convolutional network with autoencoder for spatial domain identification using multi-scale adaptation
Source: Genome Biol. 2025 Jun 10;26:159. doi: 10.1186/s13059-025-03637-z (PMC12150536; doi:10.1186/s13059-025-03637-z)
Supplement: Supplementary file 1 — Additional file 1: Fig. S1-S28. [file 13059_2025_3637_MOESM1_ESM.docx]

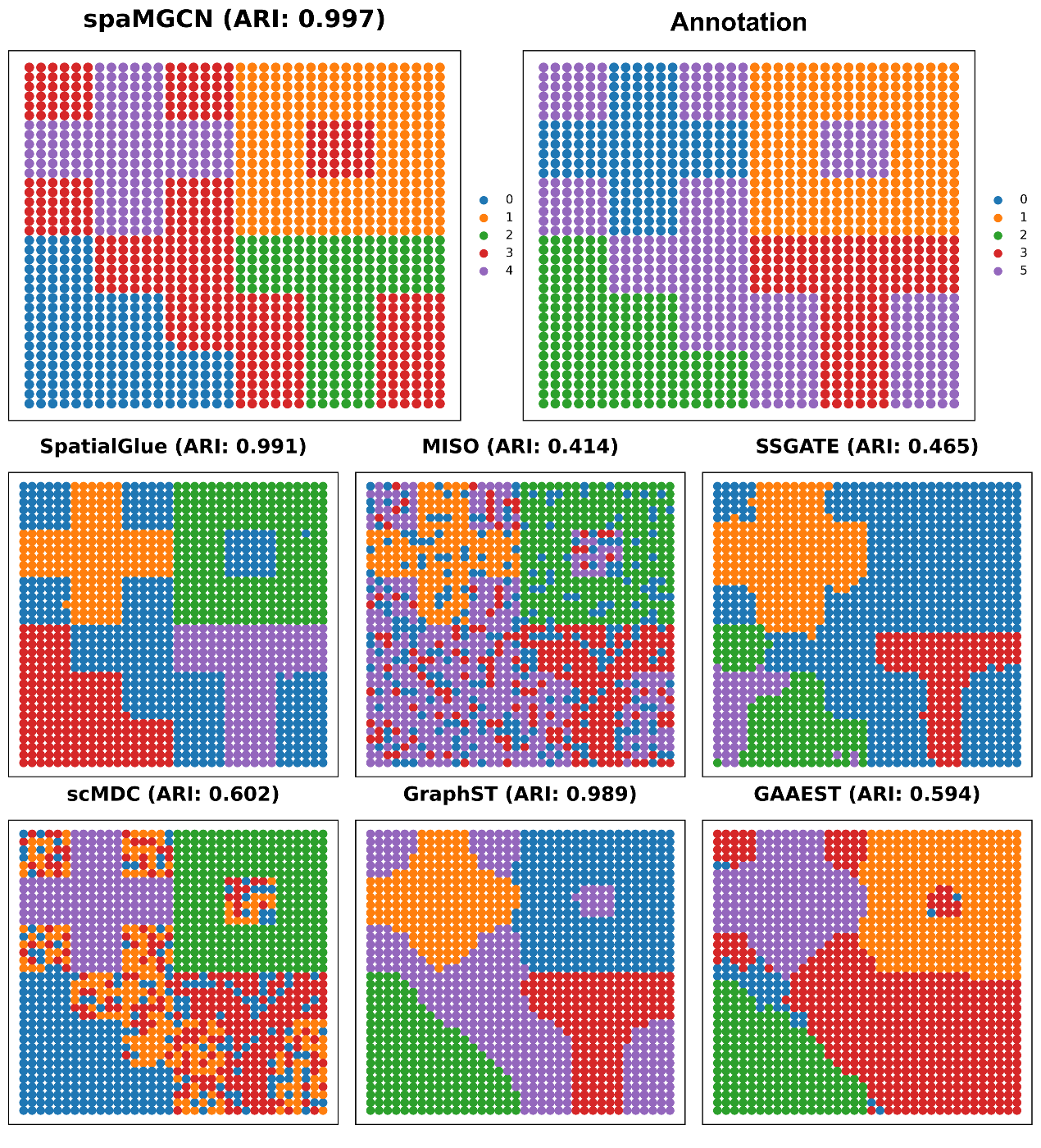


Fig. S1: Performance comparison of different methods on the simulated dataset. Note: SpaGIC failed to generate results due to NaN errors during training.


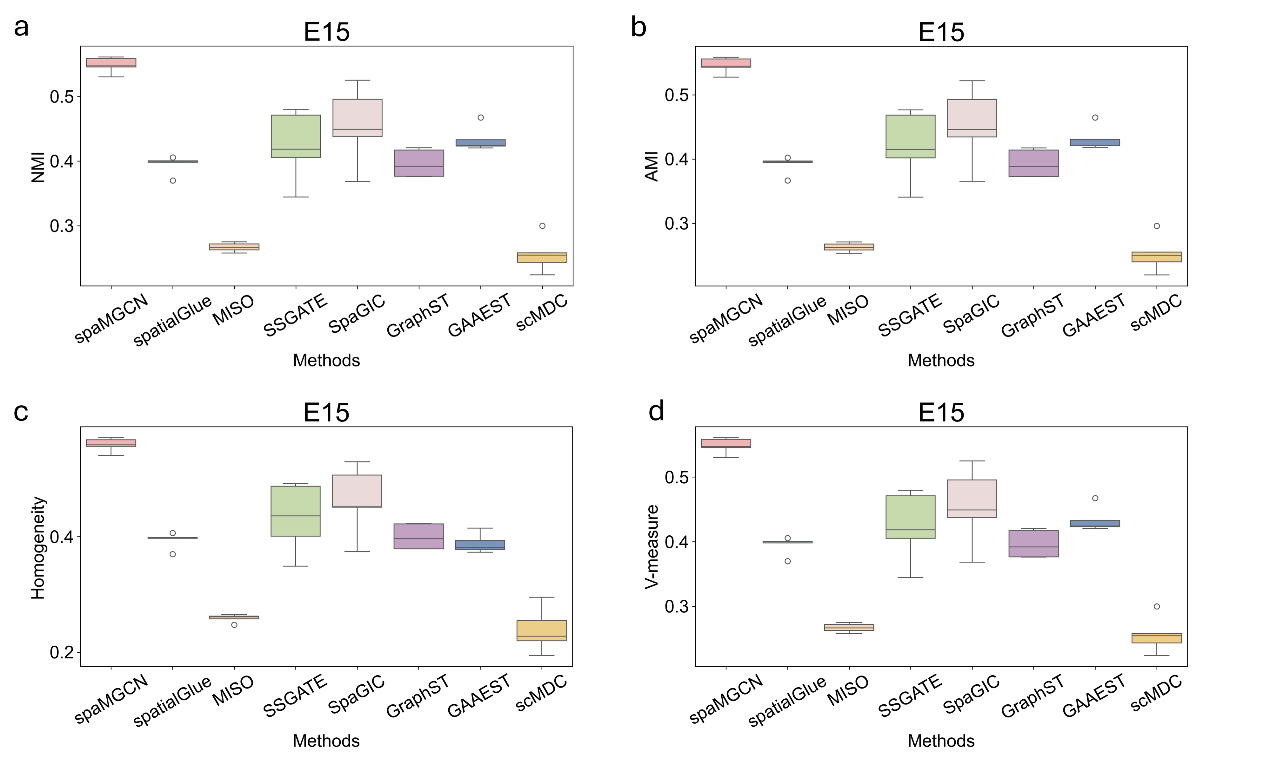


Fig. S2: Clustering performance evaluation on E15 dataset. (a) NMI performance comparison across methods. (b) AMI performance comparison across methods. (c)Homogeneity score comparison across methods. (d) V-measure comparison across methods.


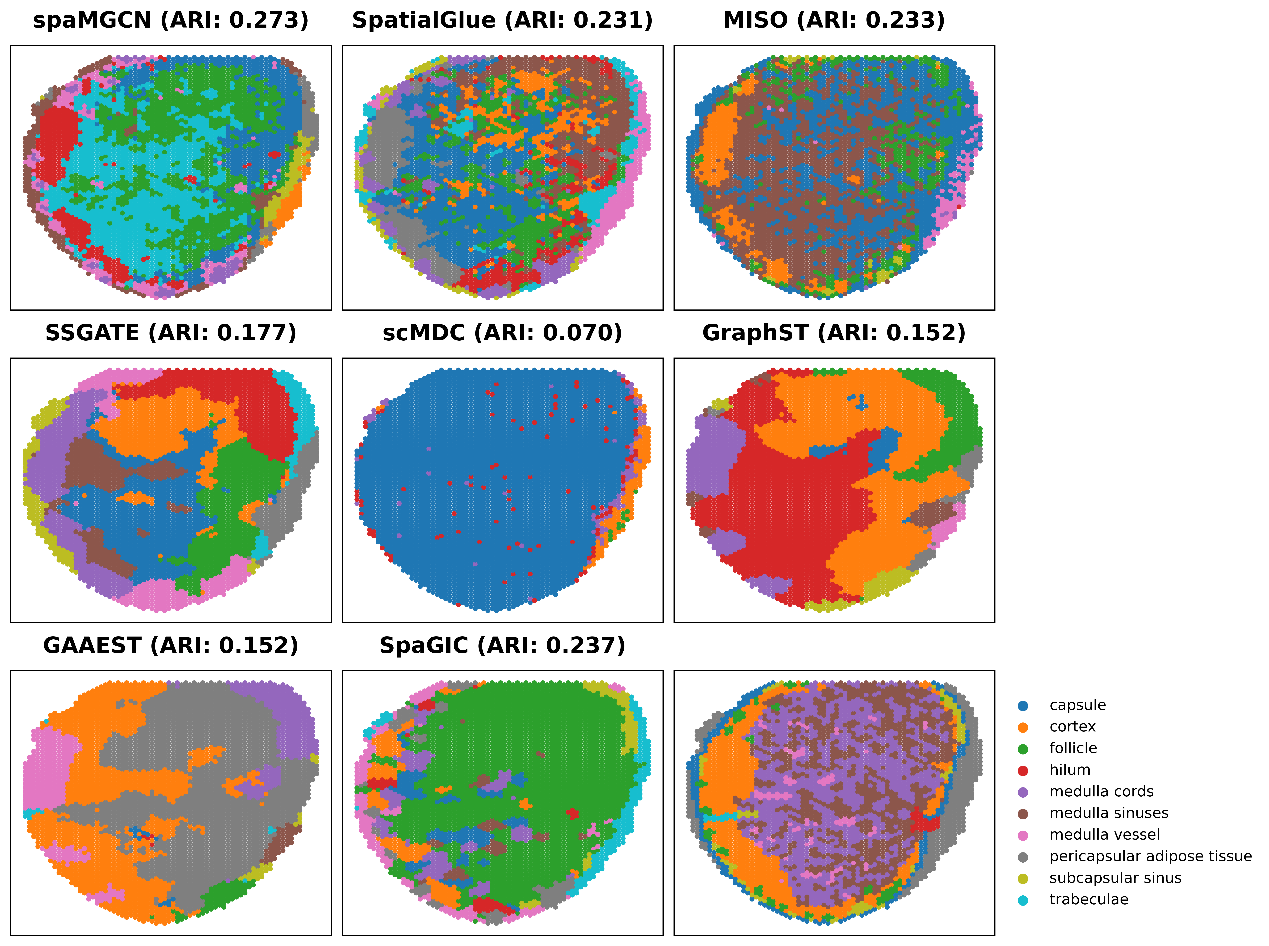


Fig. S3: Performance comparison of different methods on the human lymph node S2 dataset.


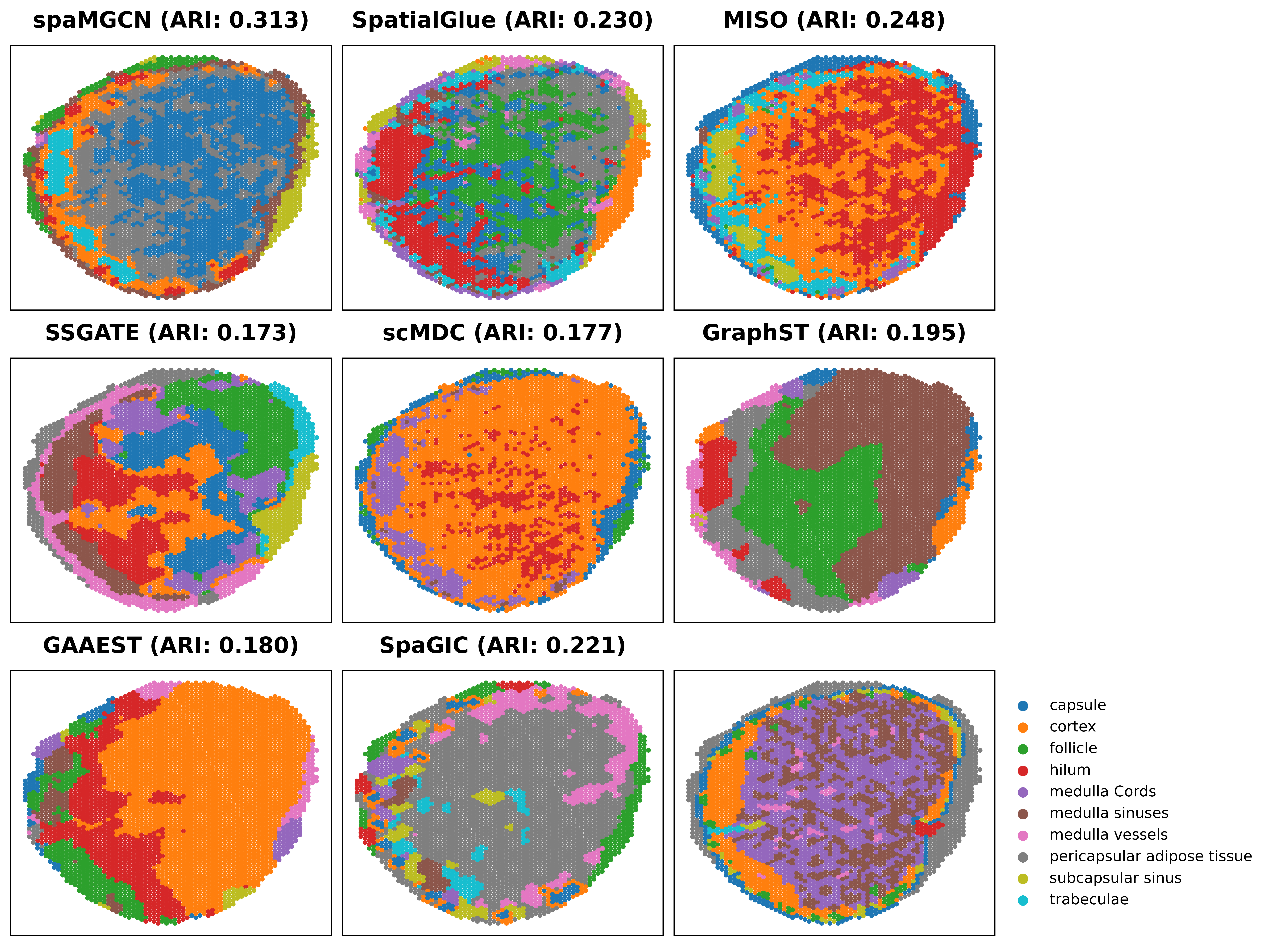


Fig. S4: Performance comparison of different methods on the human lymph node S3 dataset.


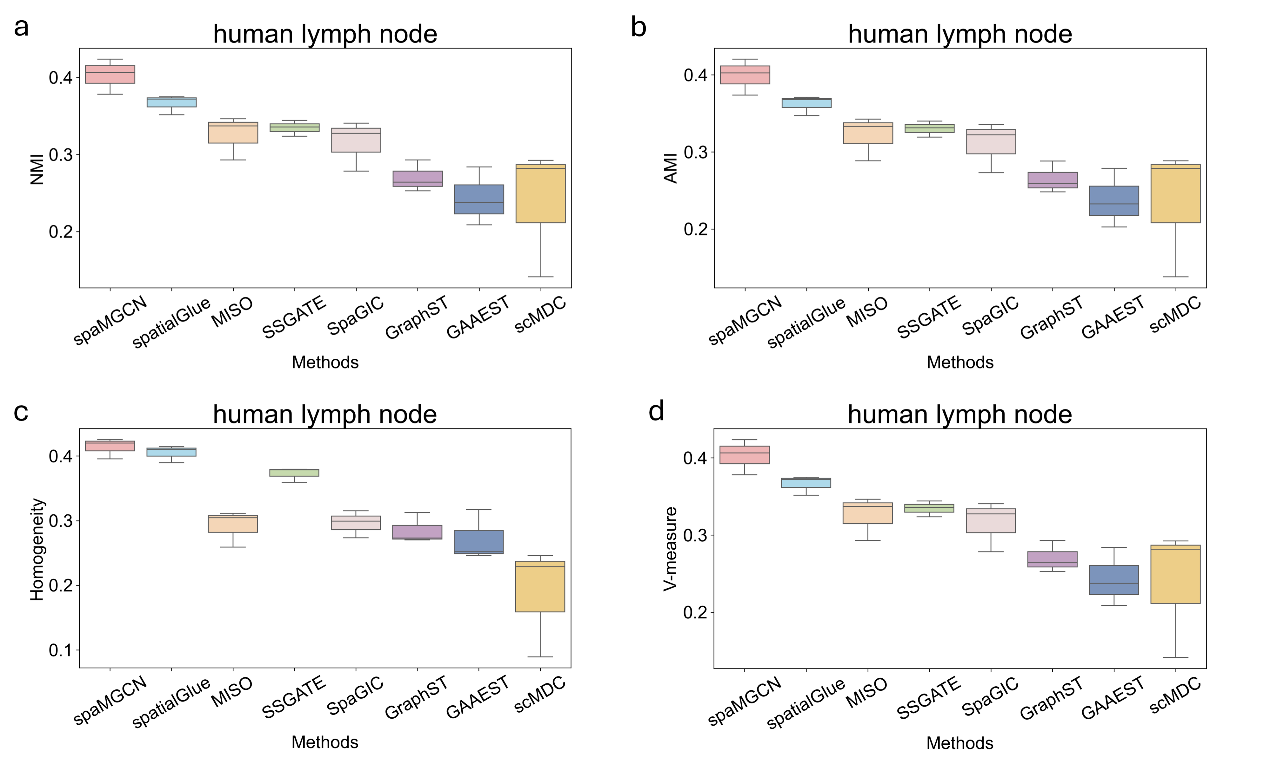


Fig. S5: Clustering performance evaluation on human lymph node dataset. (a) NMI performance comparison across methods. (b) AMI performance comparison across methods. (c) Homogeneity score comparison across methods. (d) V-measure comparison across methods.


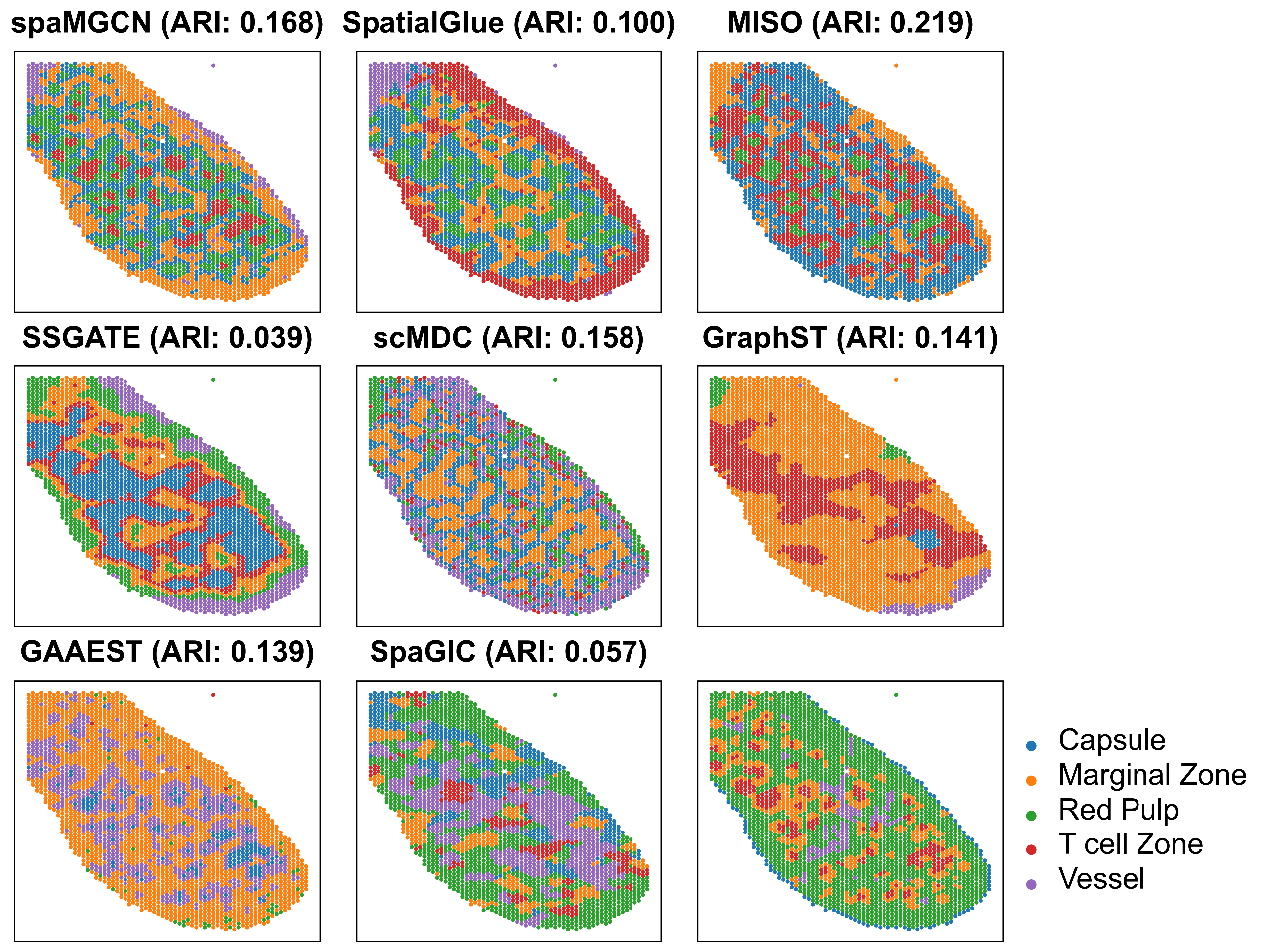


Fig. S6: Performance comparison of different methods on the spleen dataset.


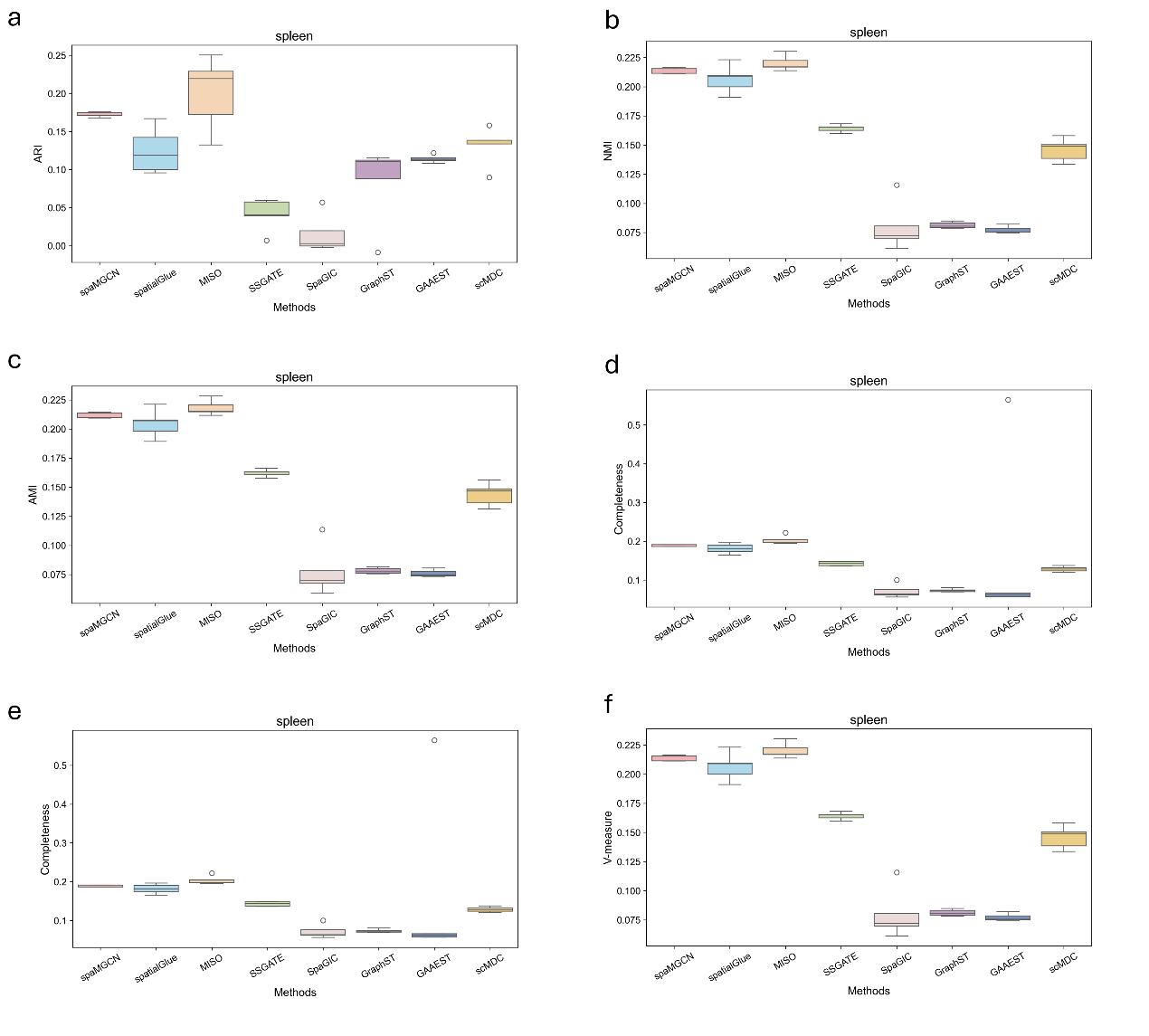


Fig. S7: Clustering performance evaluation on spleen dataset. (a) ARI performance comparison across methods. (b) NMI performance comparison across methods. (c) AMI performance comparison across methods. (d) Completeness comparison across methods. (e) Homogeneity score comparison across methods. (f) V-measure comparison across methods.


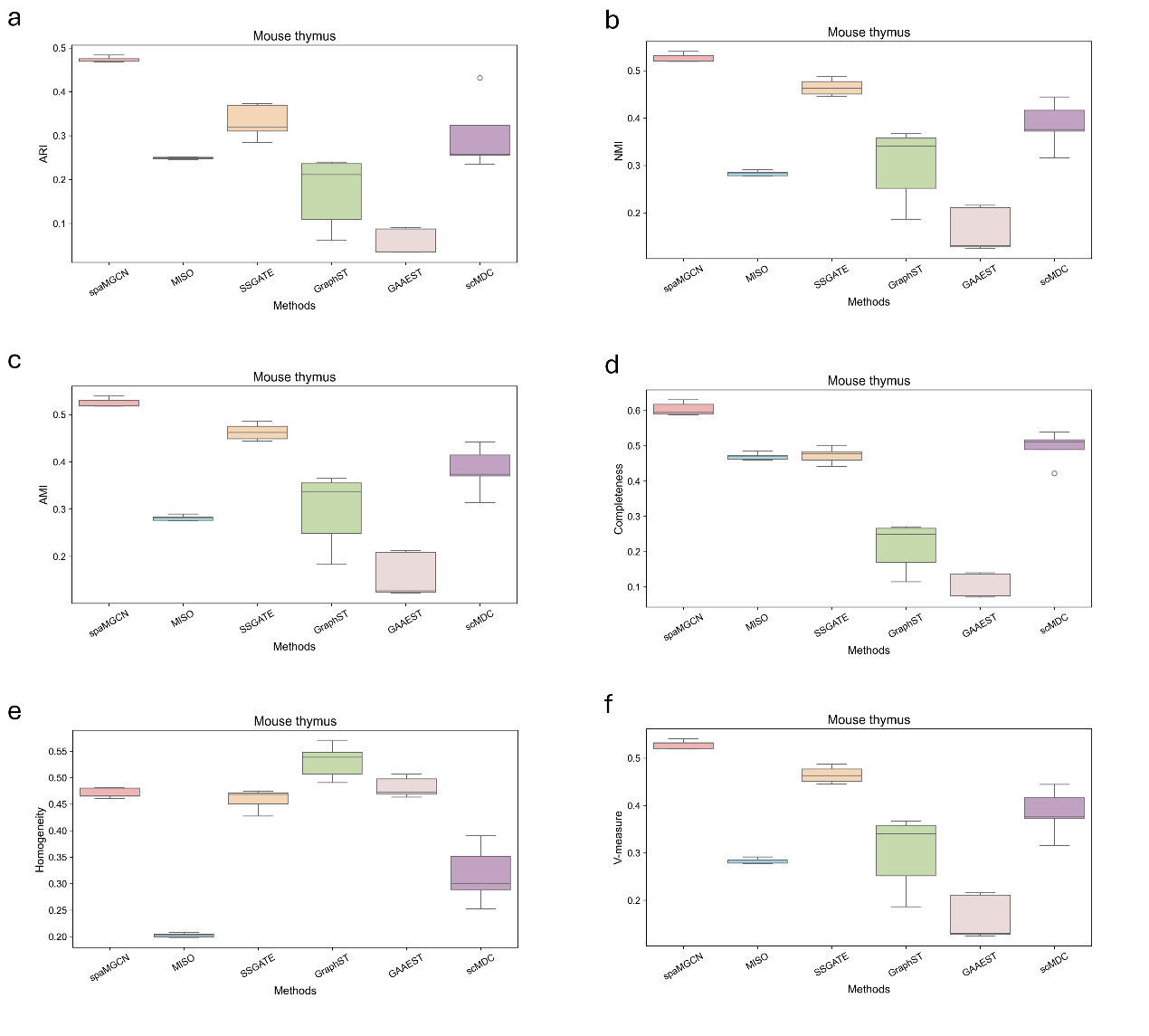


Fig. S8: Clustering performance evaluation on thymus dataset. (a) ARI performance comparison across methods. (b) NMI performance comparison across methods.

(c) AMI performance comparison across methods. (d) Completeness comparison across methods. (e) Homogeneity score comparison across methods. (f) V-measure comparison across methods.


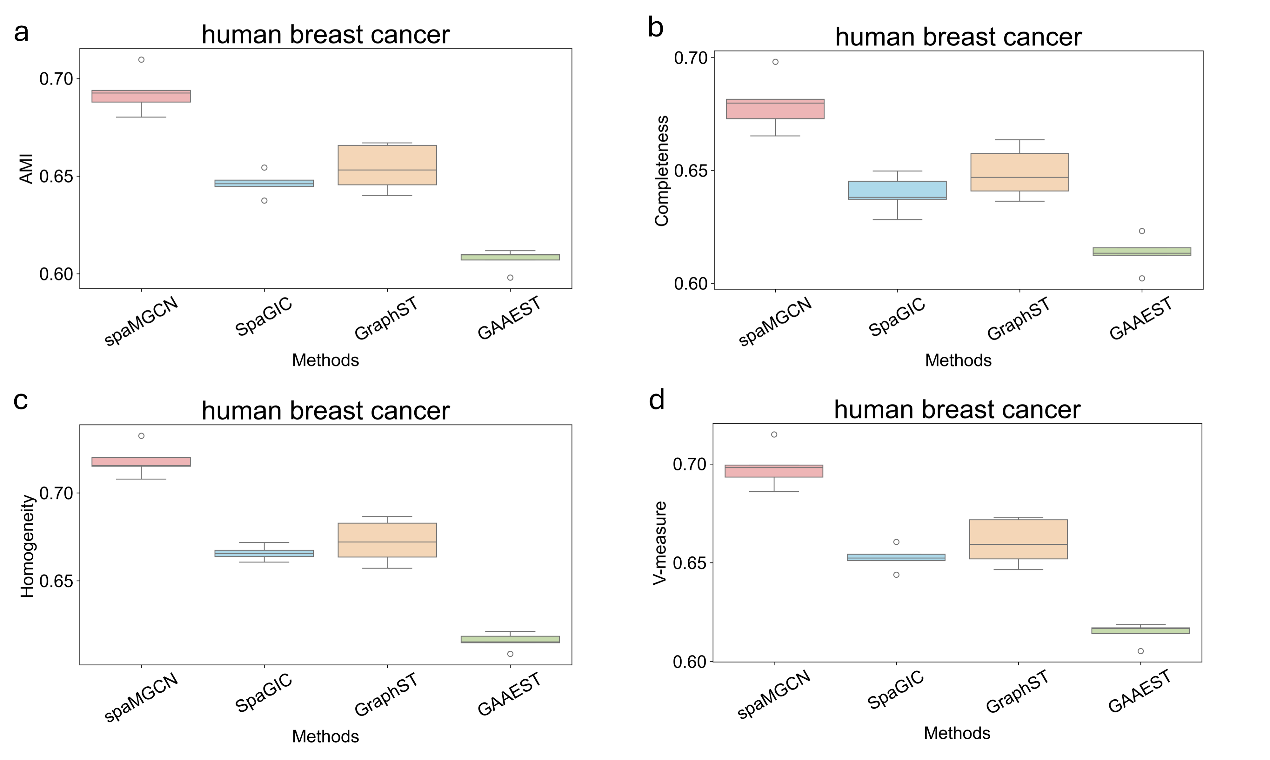


Fig. S9: Clustering performance evaluation on human breast cancer dataset. (a) AMI performance comparison across methods. (b)Completeness performance comparison across methods. (c) Homogeneity score comparison across methods. (d) V-measure comparison across methods.


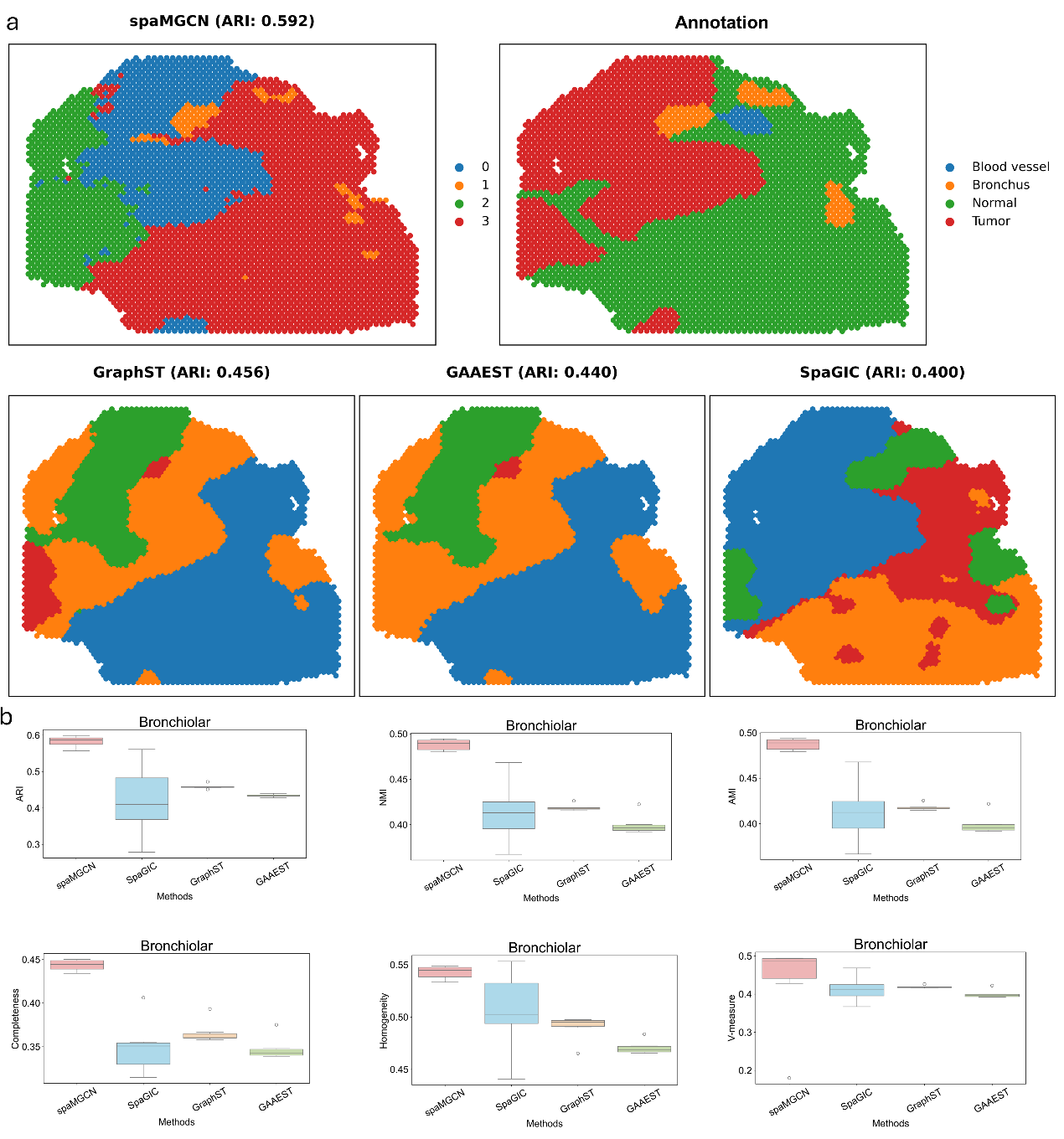


Fig. S10: Comparative analysis of spatial domain segmentation and clustering performance across methods using the Bronchiolar dataset. (a) Spatial domain patterns identified by spaMGCN versus baseline methods. (b) Quantitative evaluation of clustering performance metrics.


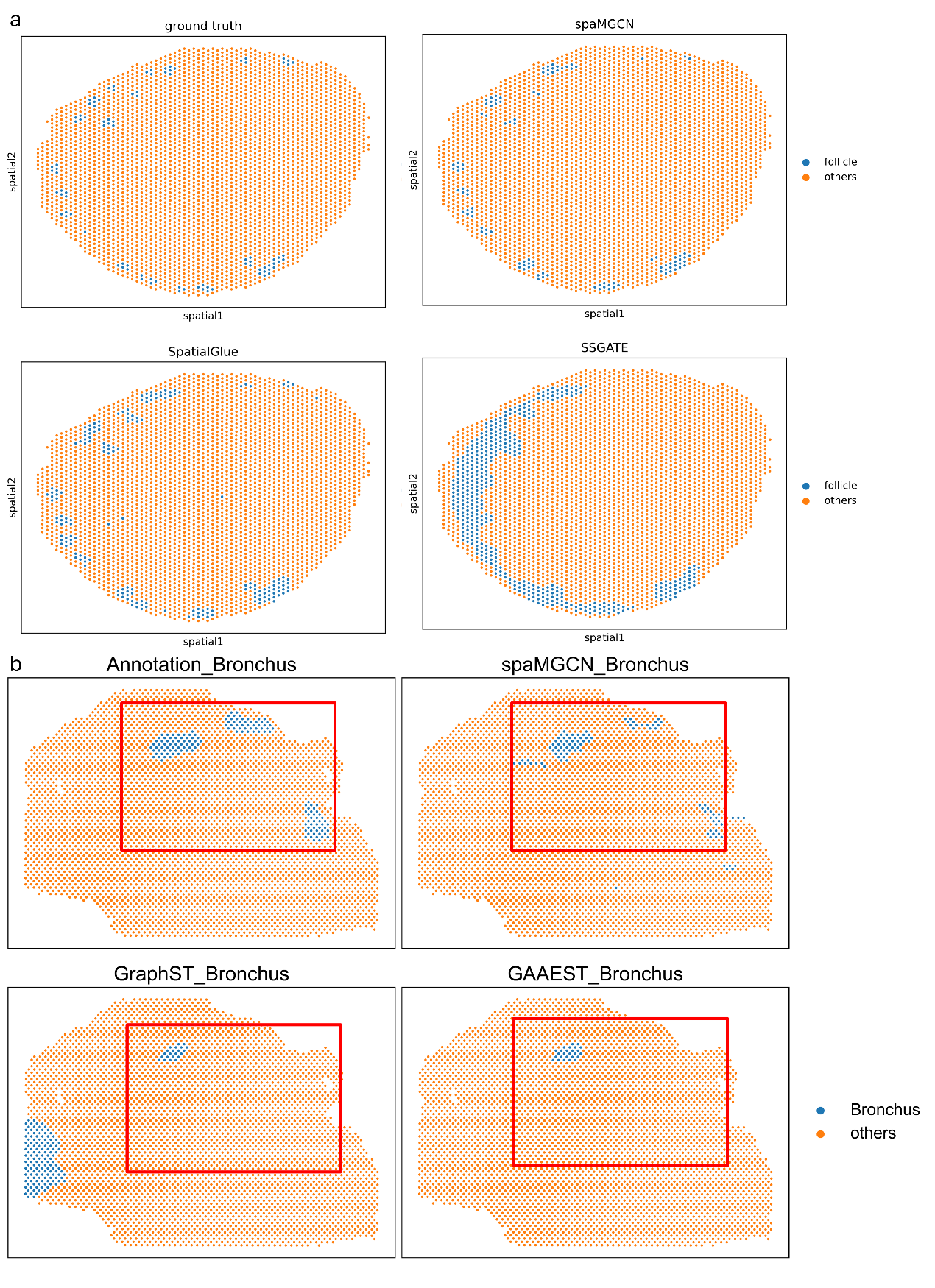


Fig. S11: Comparison of the performance of different spatial multi-omics spatial domain identification methods for recognizing discrete spatial domains.(a) Identifying follicles using different methods on the human lymph node S3 dataset (b)Identifying Bronchus using different methods on the bronchial tumors dataset


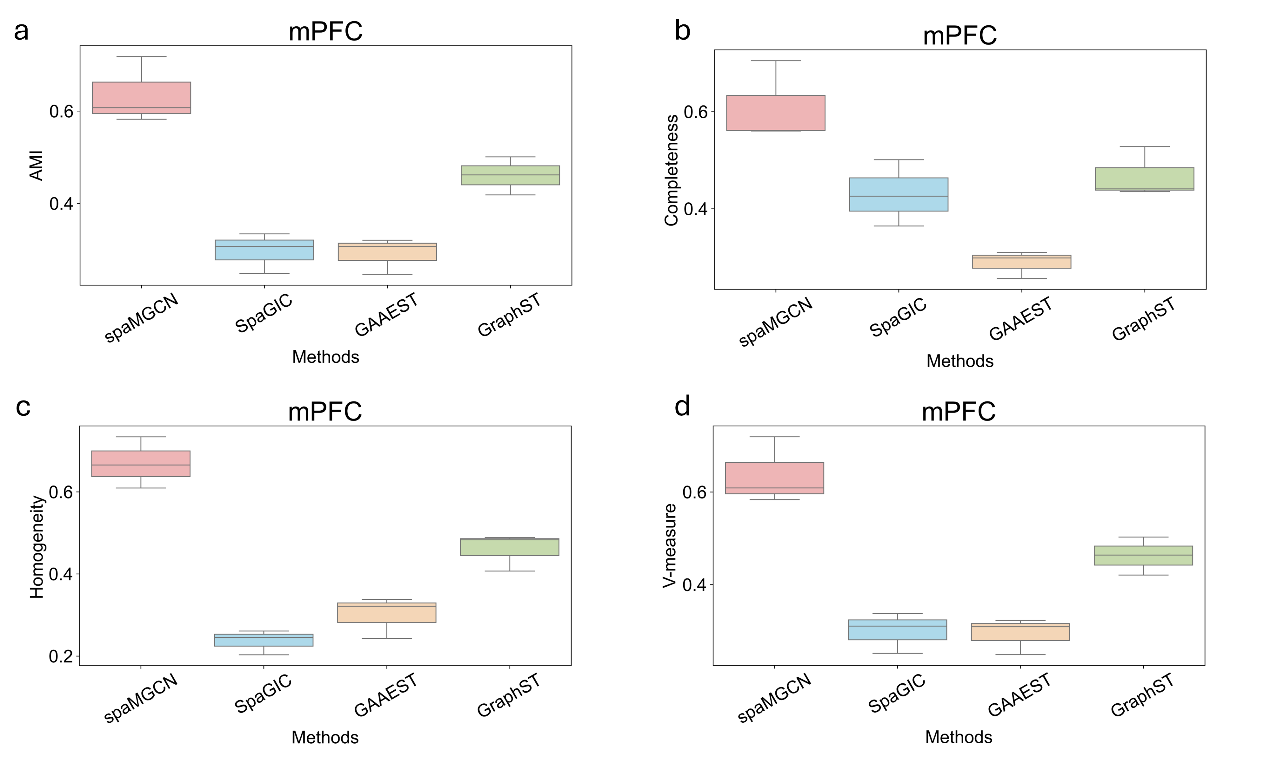


Fig. S12: Clustering performance evaluation on mPFC dataset. (a) AMI performance comparison across methods. (b)Completeness performance comparison across methods. (c) Homogeneity score comparison across methods. (d) V-measure comparison across methods.


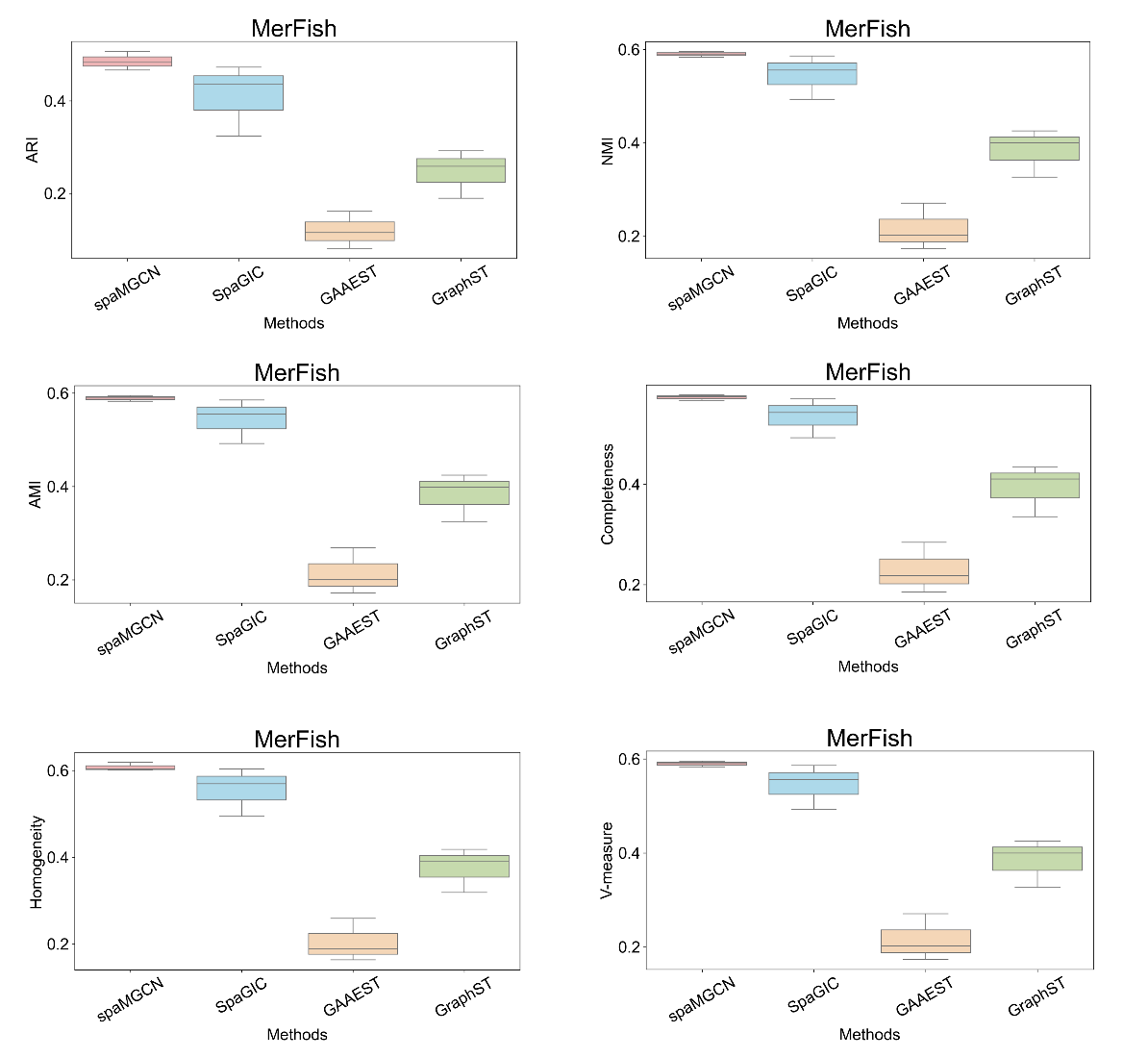


Fig. S13: Clustering performance evaluation on MerFish dataset. (a) ARI performance comparison across methods. (b) NMI performance comparison across methods. (c) AMI performance comparison across methods. (d) Completeness comparison across methods. (e) Homogeneity score comparison across methods. (f) V-measure comparison across methods.


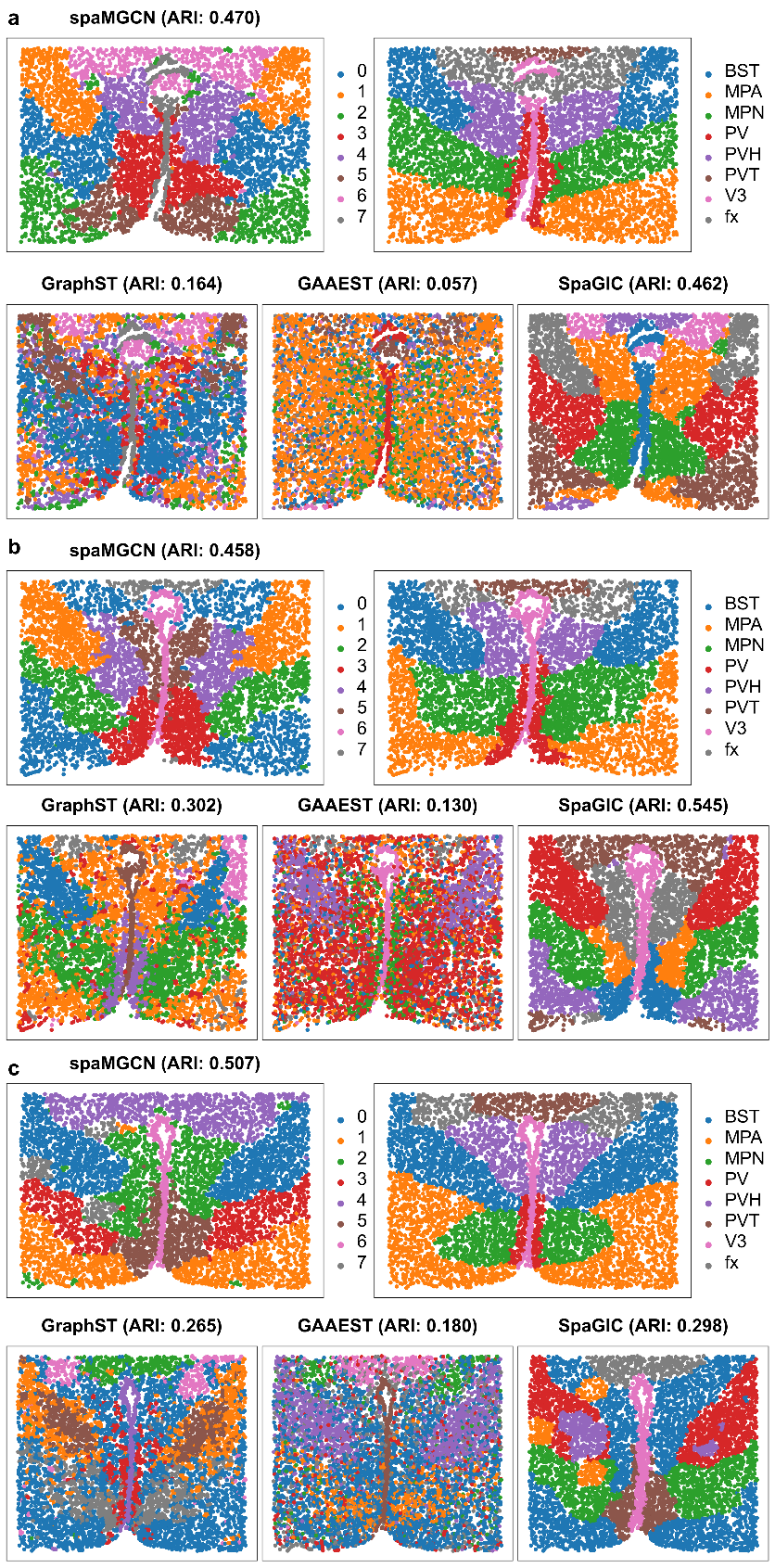


Fig. S14: Visualization of spatial domains identified by different methods on MERFISH data. (a) Spatial domain segmentation results across methods for the merfish0.04 dataset. (b) Spatial domain segmentation results across methods for the merfish0.09 dataset. (c) Spatial domain segmentation results across methods for the merfish0.14 dataset.


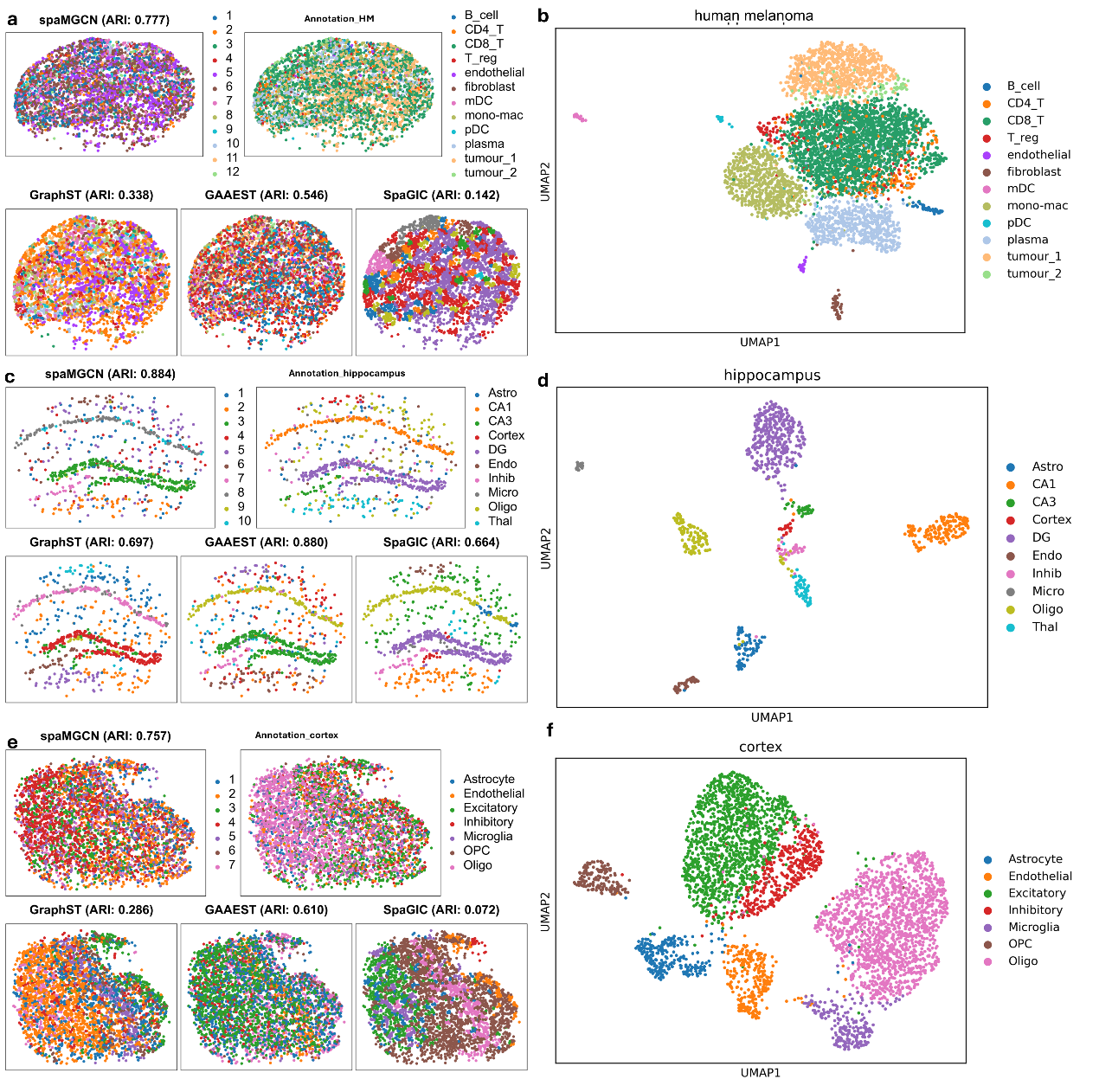


Fig. S15: Clustering results of different methods across multiple single-cell resolution spatial transcriptomics datasets. (a) Visualization of clustering performance by different methods on the human melanoma dataset. (b) UMAP plot of features extracted by spaMGCN for the human melanoma dataset. (c) Clustering visualization of different methods on the hippocampus dataset. (d) UMAP plot of features extracted by spaMGCN for the hippocampus dataset. (e) Clustering performance comparison across methods on the human cortex dataset. (f) Feature UMAP visualization generated by spaMGCN for the human cortex dataset


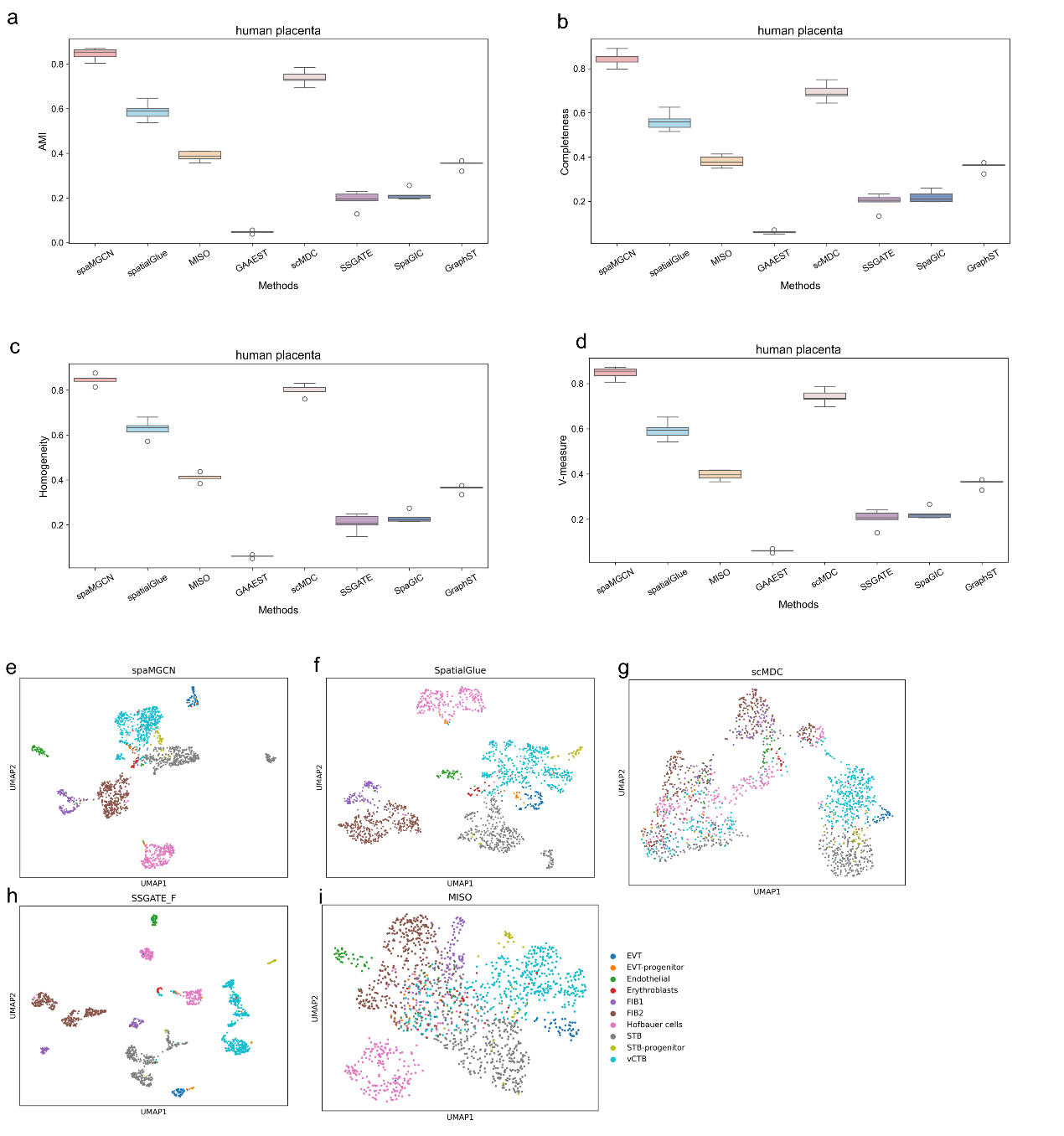


Fig. S16: Clustering performance and feature space visualization of different methods on the human placental dataset. (a) Adjusted Mutual Information (AMI) scores across methods. (b) Completeness metric comparison. (c) Homogeneity score evaluation. (d) V-measure performance analysis. (e-i) UMAP visualizations of features extracted by each respective method


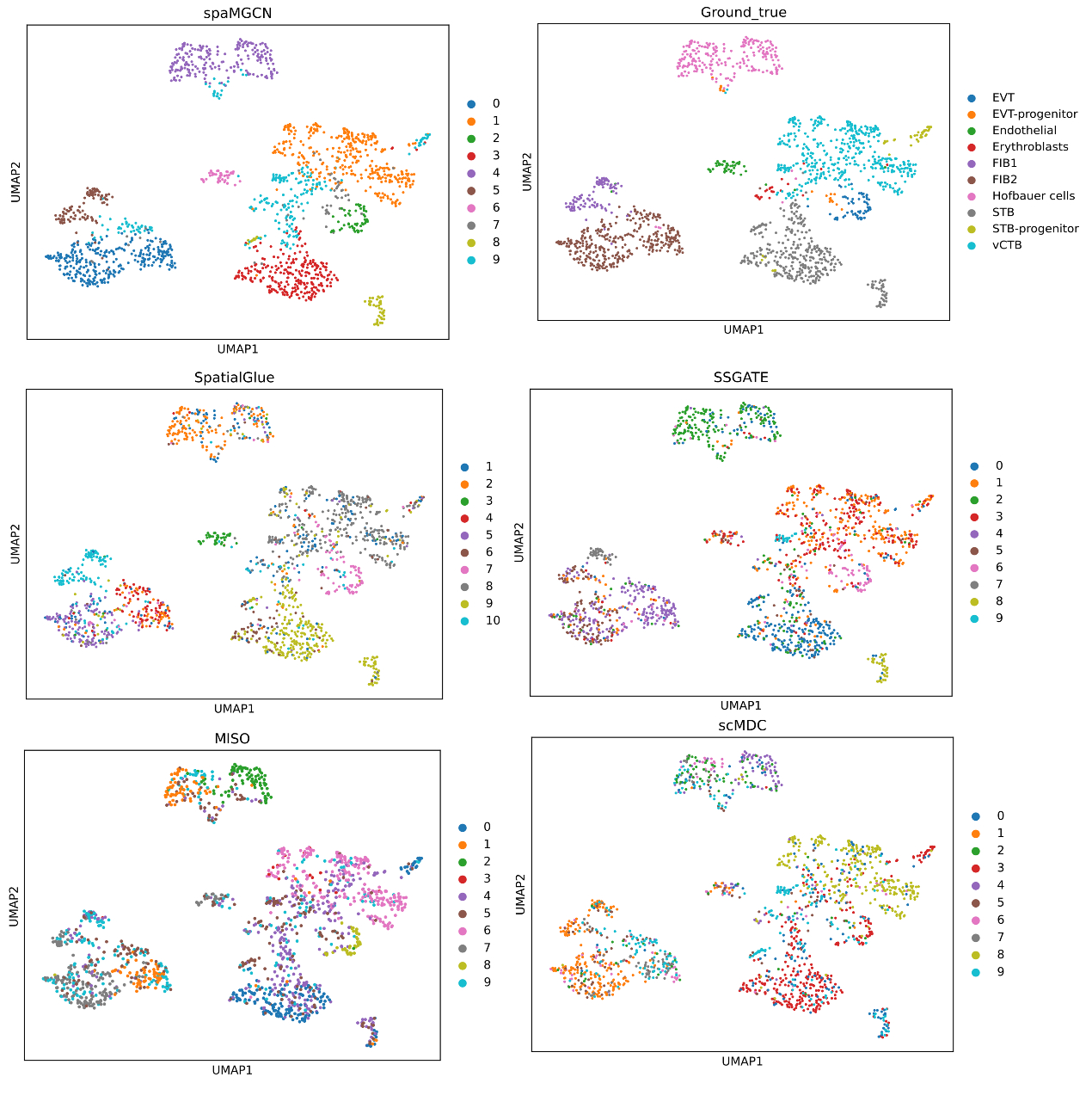


Fig. S17: Visual representation of clustering results from different methods compared to the ground truth in the same feature space.


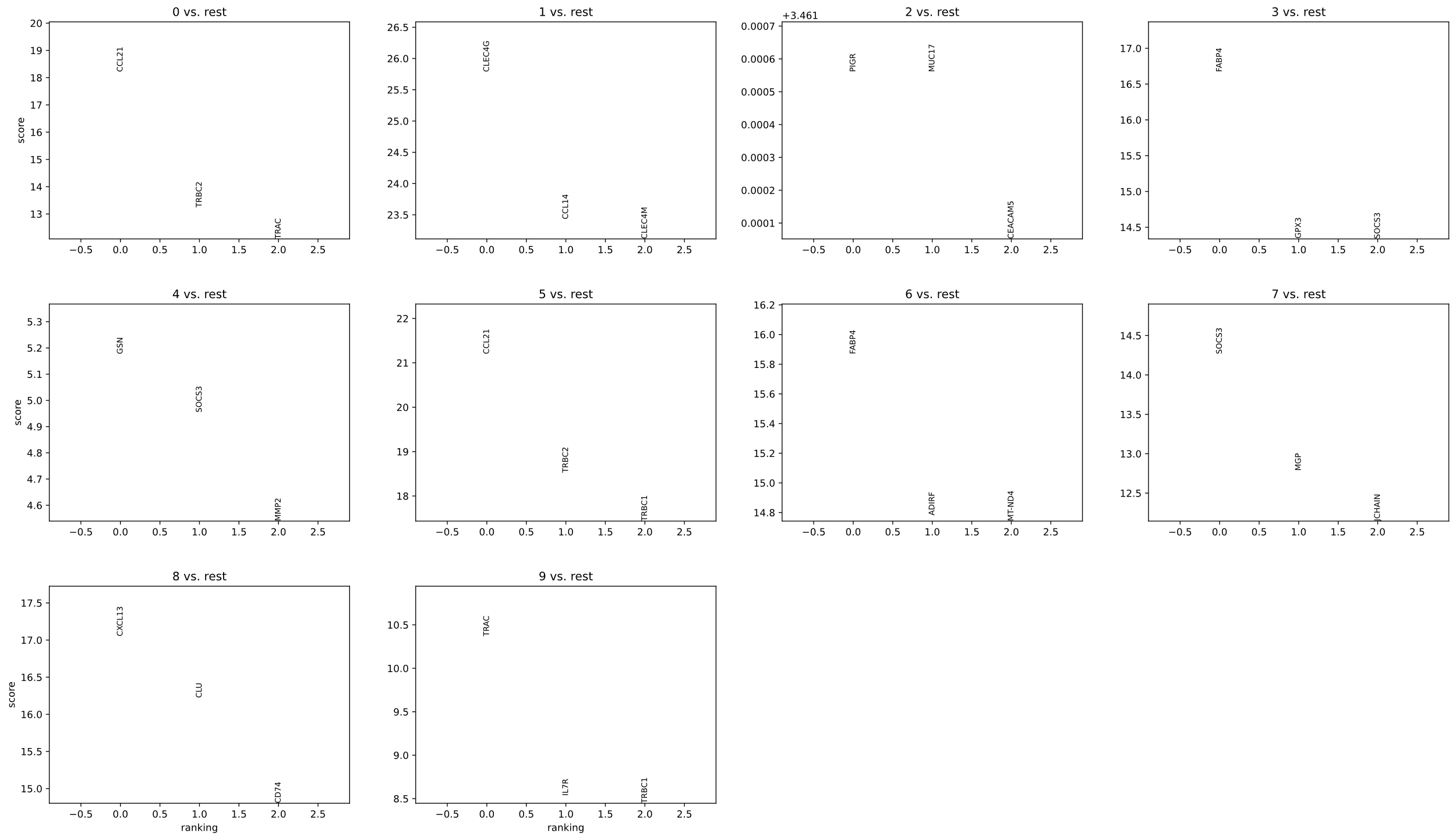


Fig. S18: Differentially expressed genes identified from spaMGCN's spatial domains in the human lymph node S1 dataset


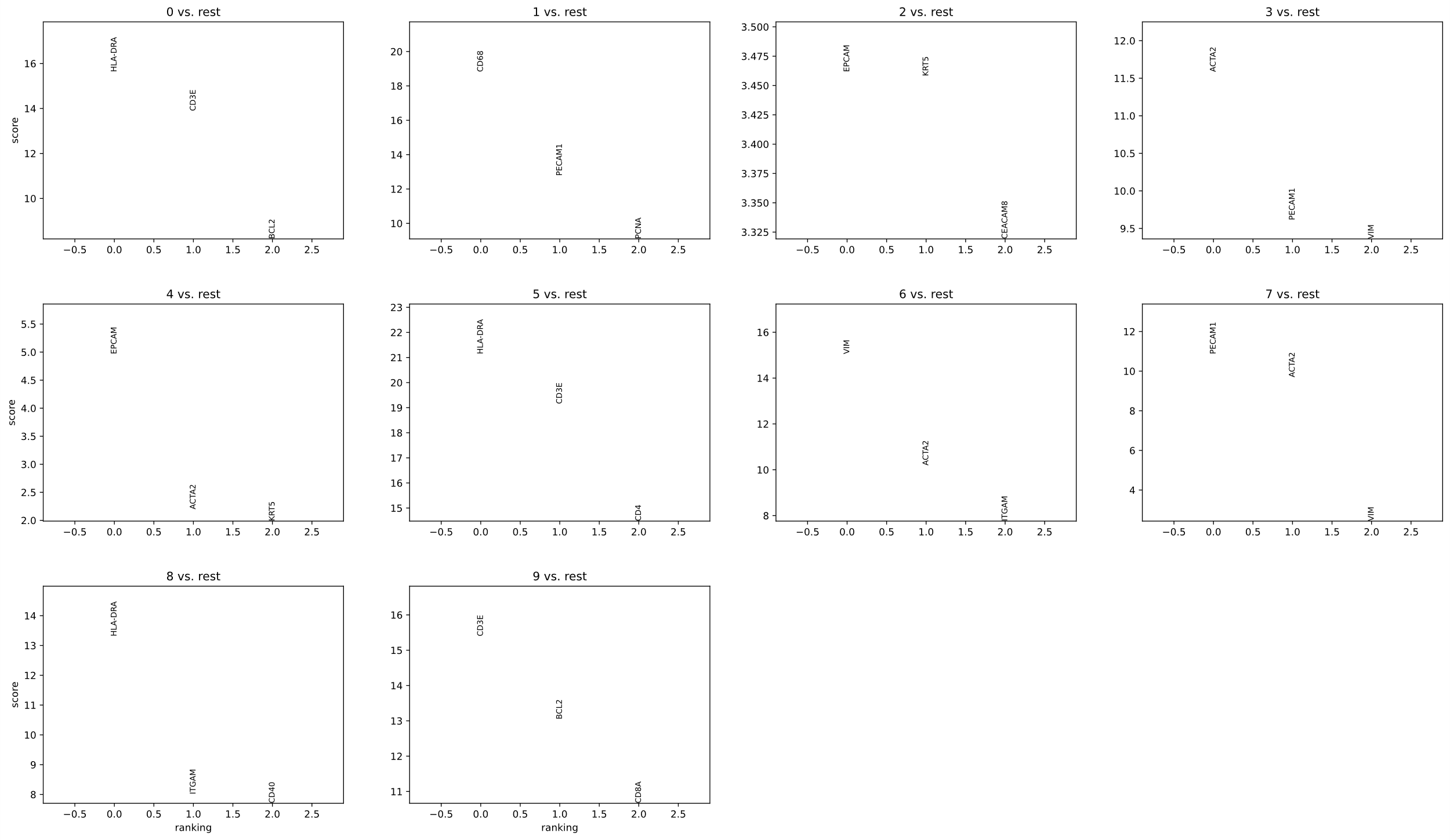


Fig. S19: Differential protein presentation based on the spatial domain partitioning results of spaMGCN in the human lymph node S1 dataset.


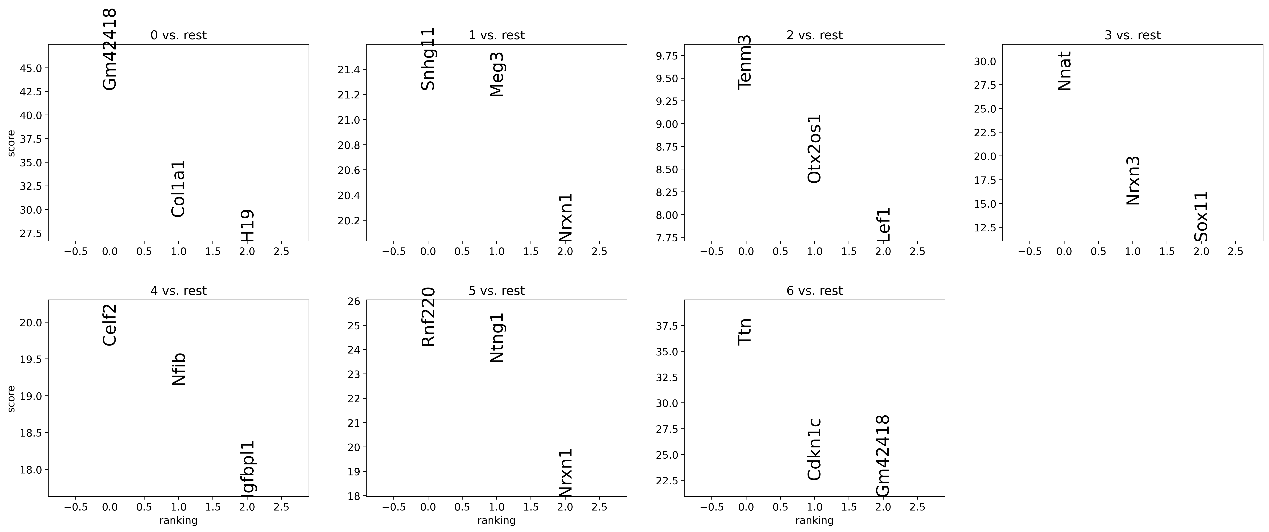


Fig. S20: Differentially expressed genes identified from spaMGCN's spatial domains in the E15 dataset


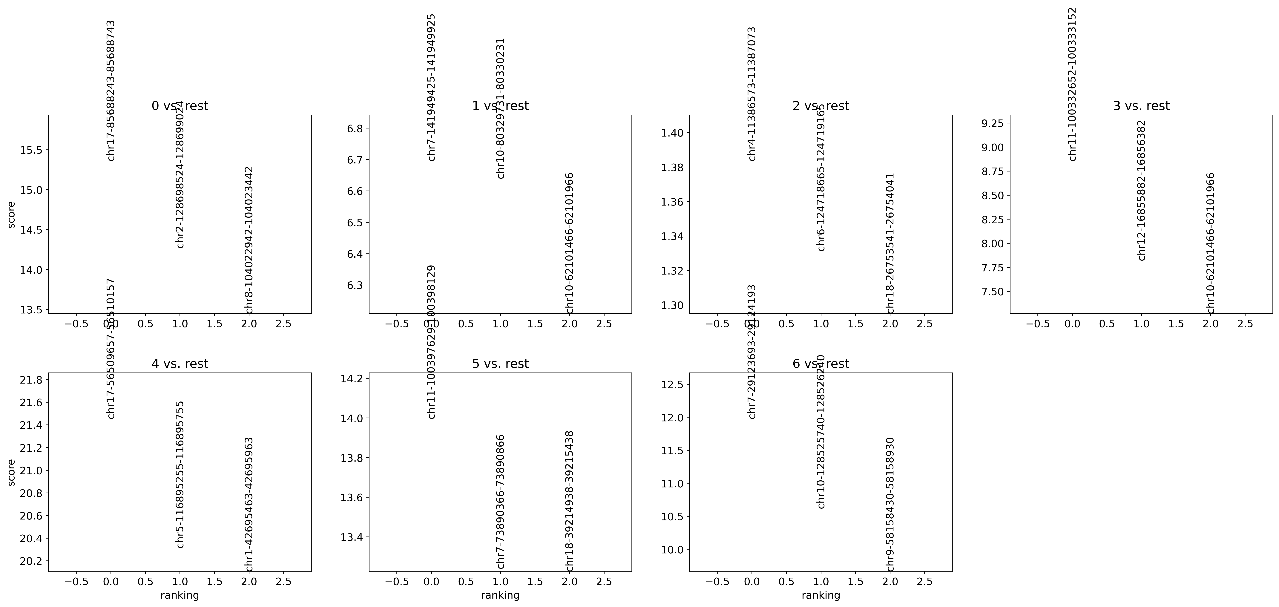


Fig. S21: Differential chromatin accessibility regions identified from spaMGCN's spatial domains in the E15 mouse embryonic dataset.


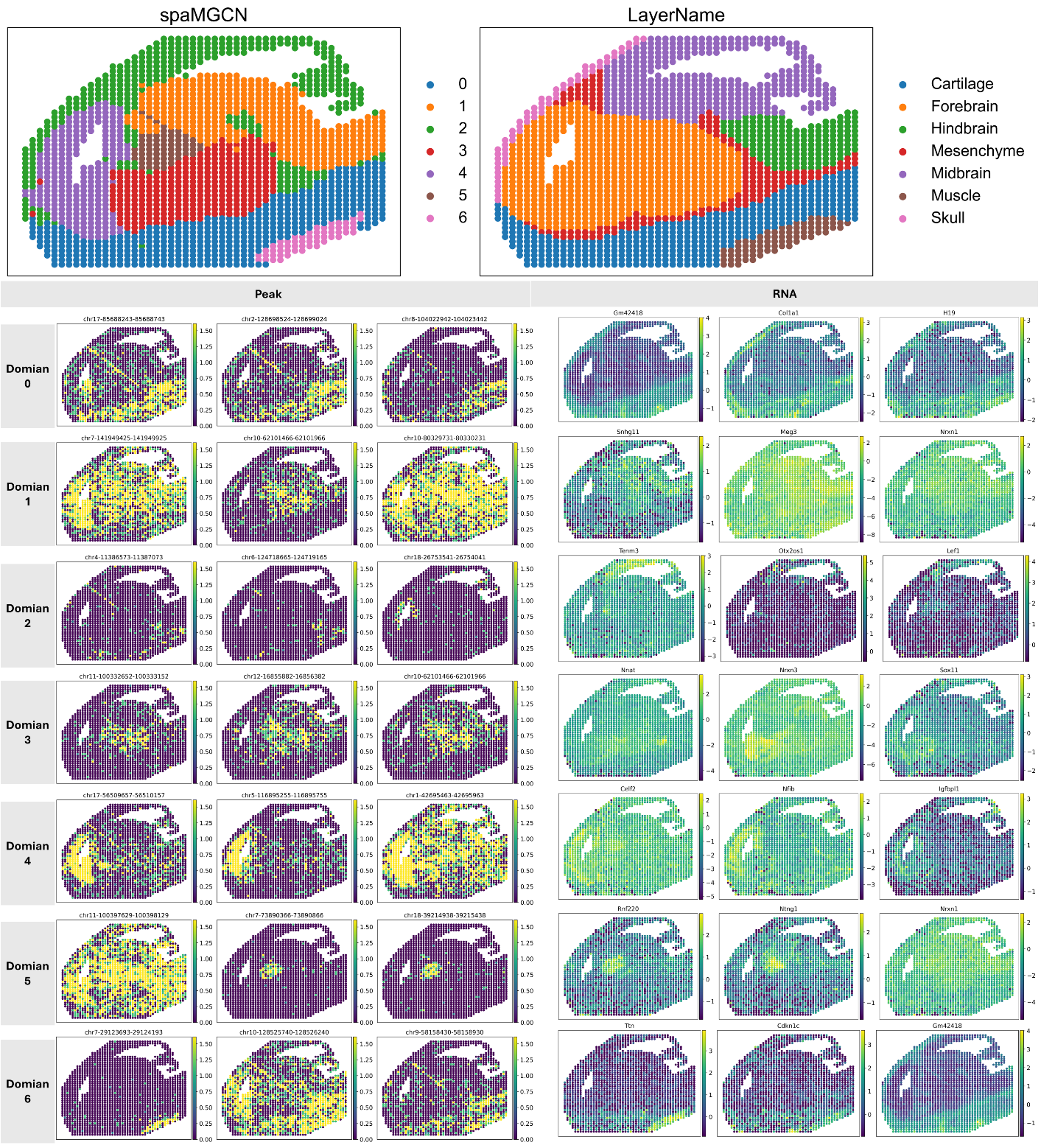


Fig. S22: Differentially expressed genes and chromatin accessible regions across spaMGCN-identified spatial domains in the E15 dataset can be visualized.


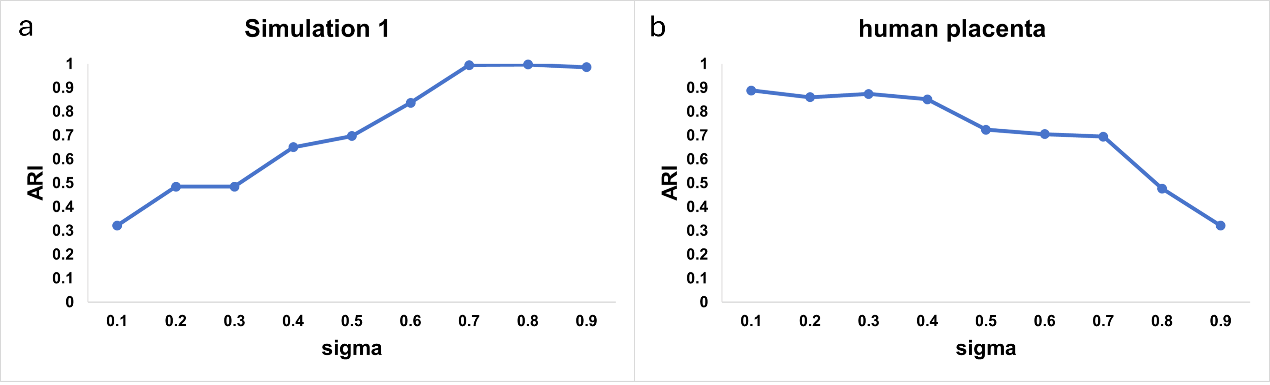


Fig. S23： Performance comparison of spaMGCN under different sigma values. (a) Evaluation on the simulated dataset across varying sigma parameters. (b) Performance assessment on the human placental dataset at different sigma settings.


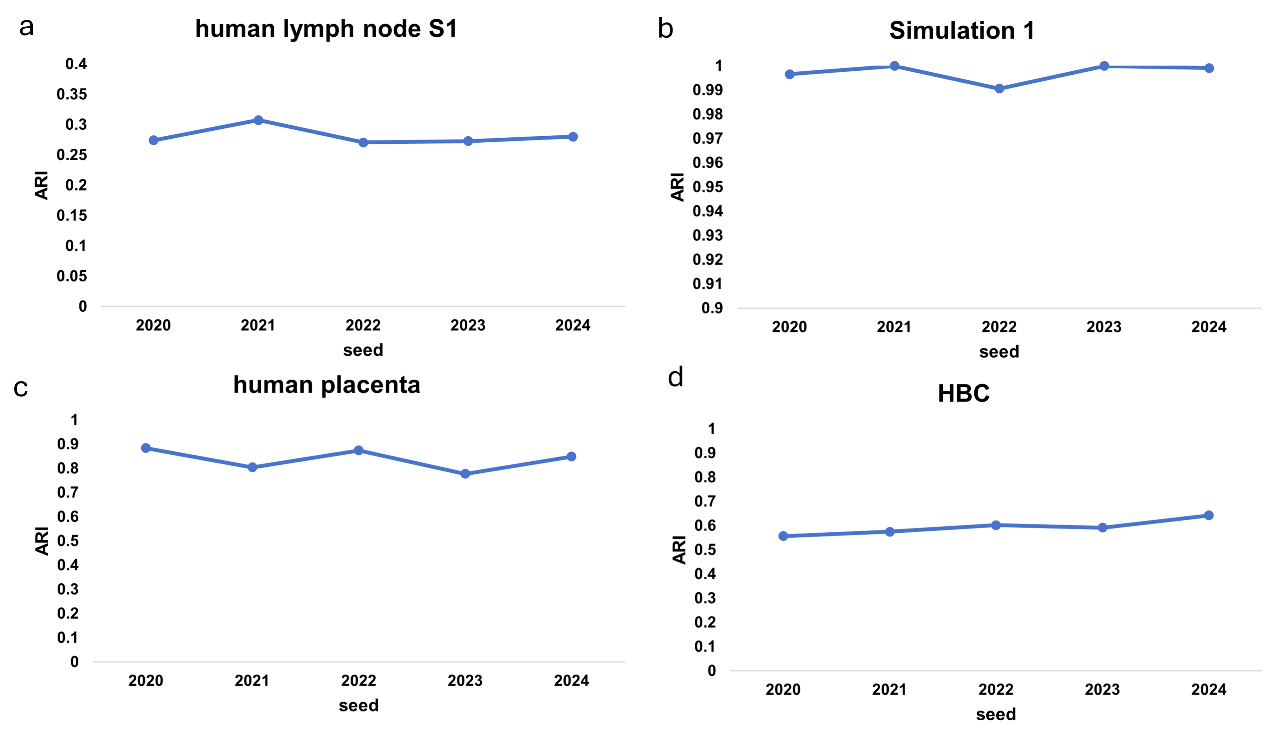


Fig. S24: Performance comparison of spaMGCN under different random seed. (a) Evaluation on the S1 dataset across varying random seed. (b) Performance assessment on the simulated dataset at different random seed settings. (c) Performance assessment on the human placental dataset at different random seed settings. (d) Performance assessment on the Human Breast Cancer dataset at different random seed settings.


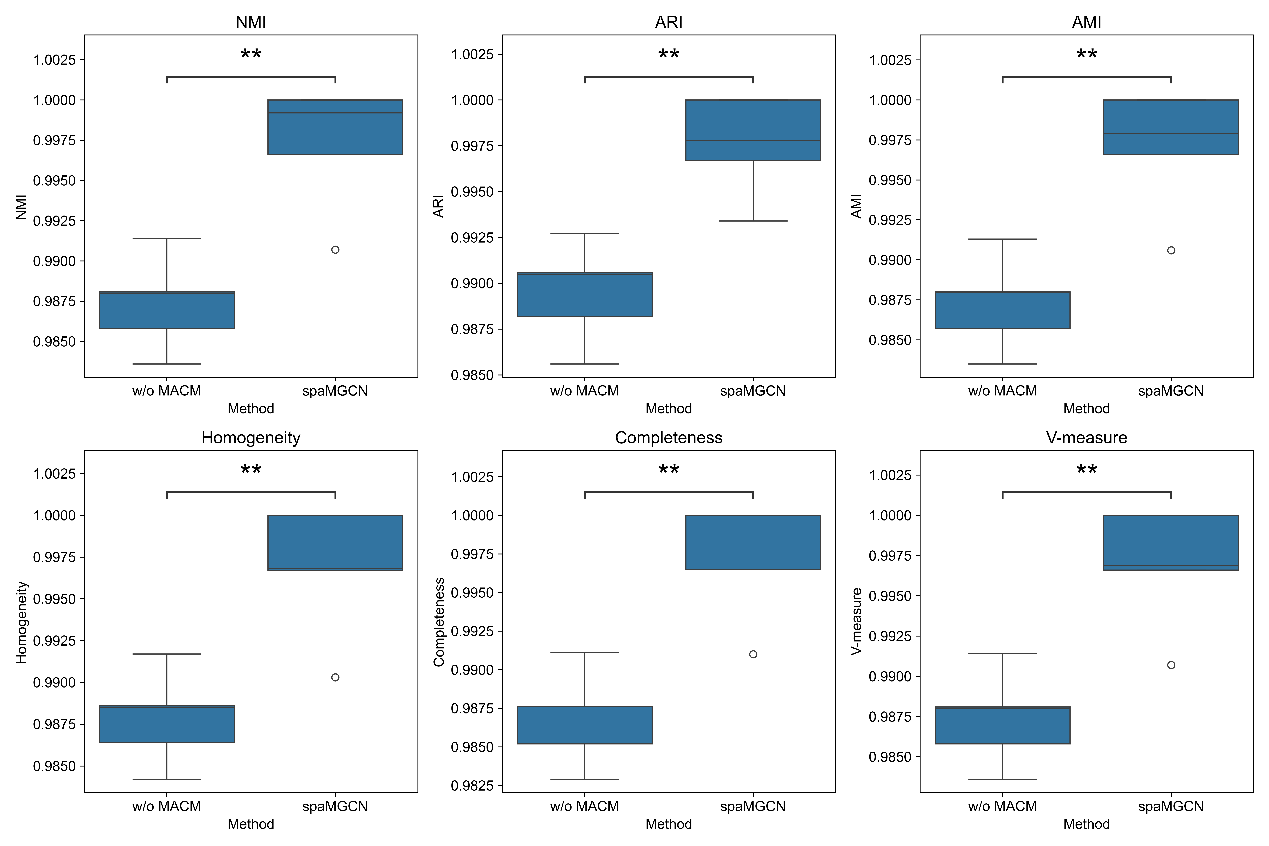


Fig. S25: Comparison of clustering performance between spaMGCN and its variant (w/o MACM) on the simulated dataset.


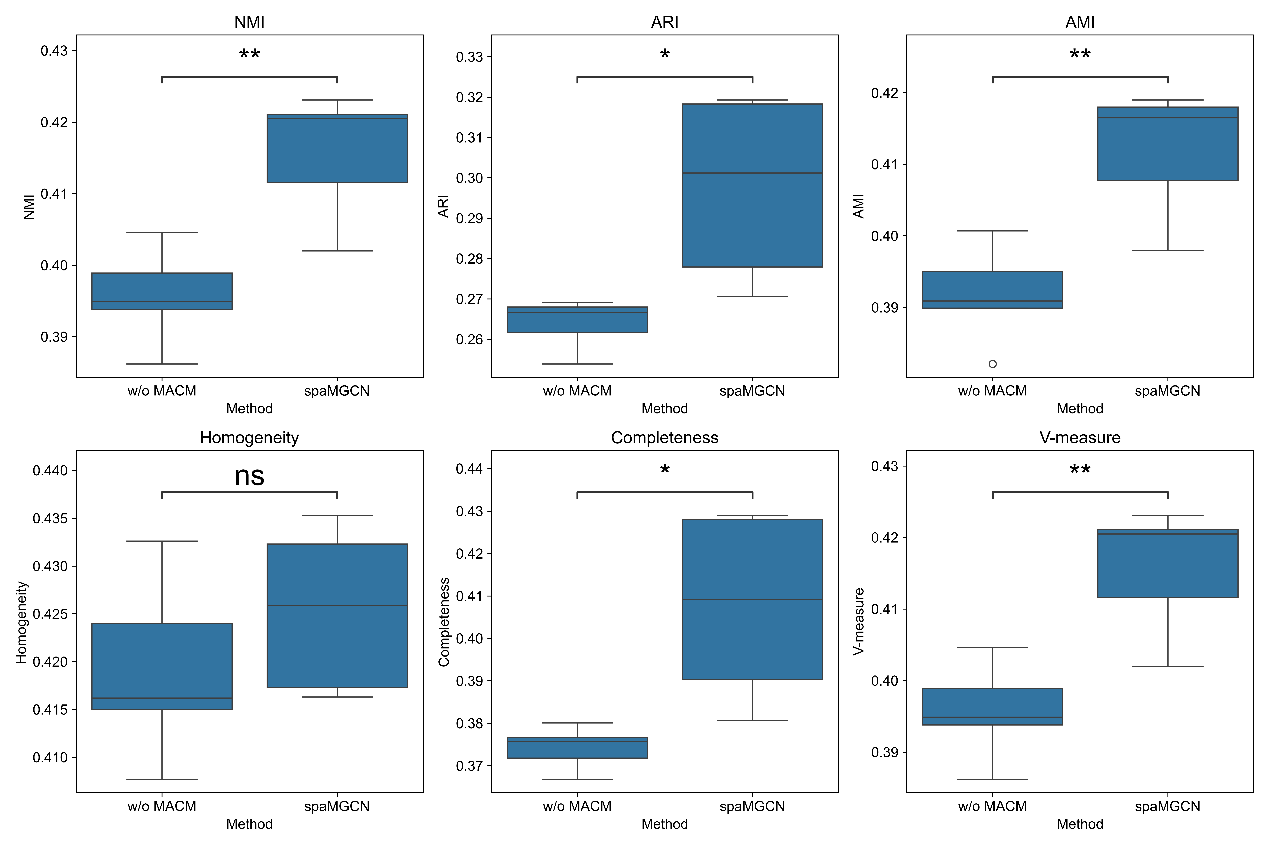


Fig. S26: Comparison of clustering performance between spaMGCN and its variant (w/o MACM) on the S1 dataset.


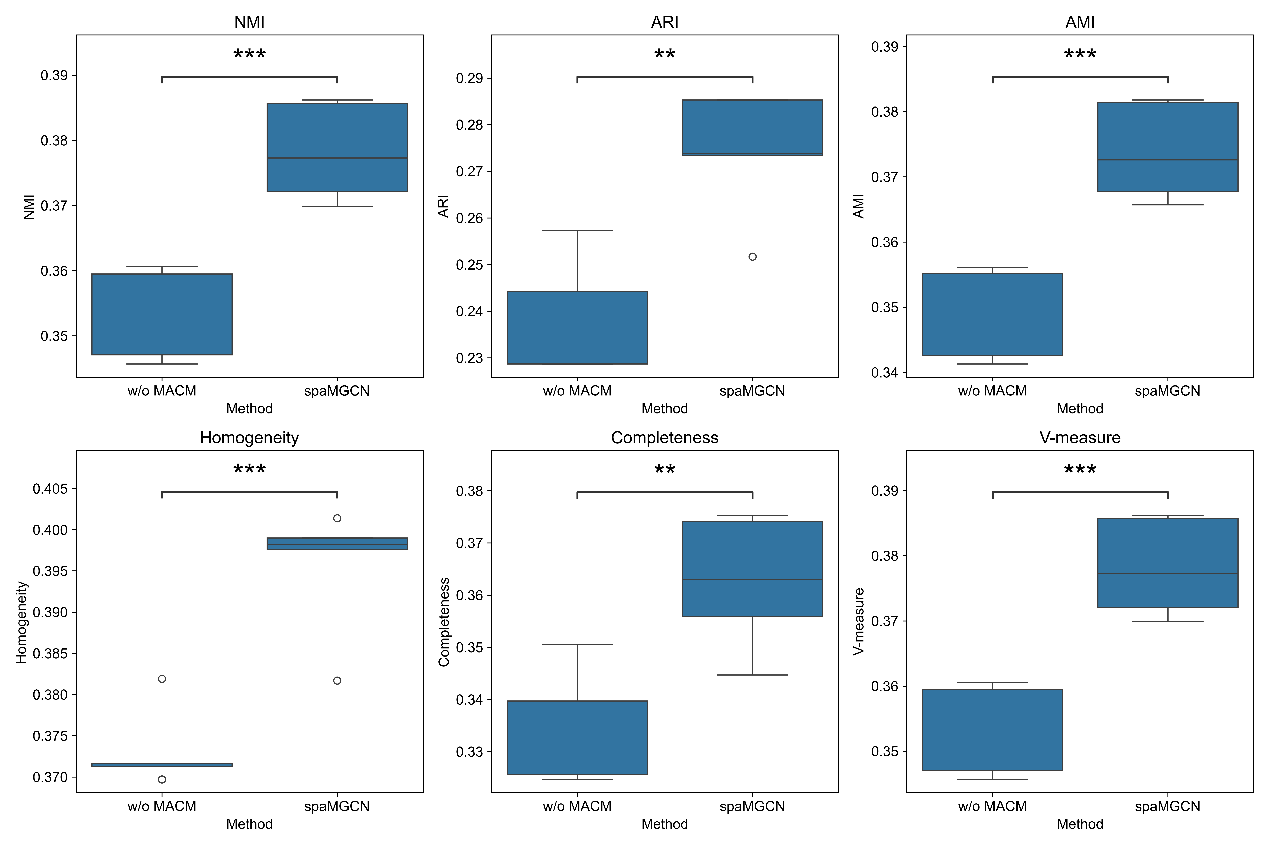


Fig. S27: Comparison of clustering performance between spaMGCN and its variant (w/o MACM) on the S2 dataset.


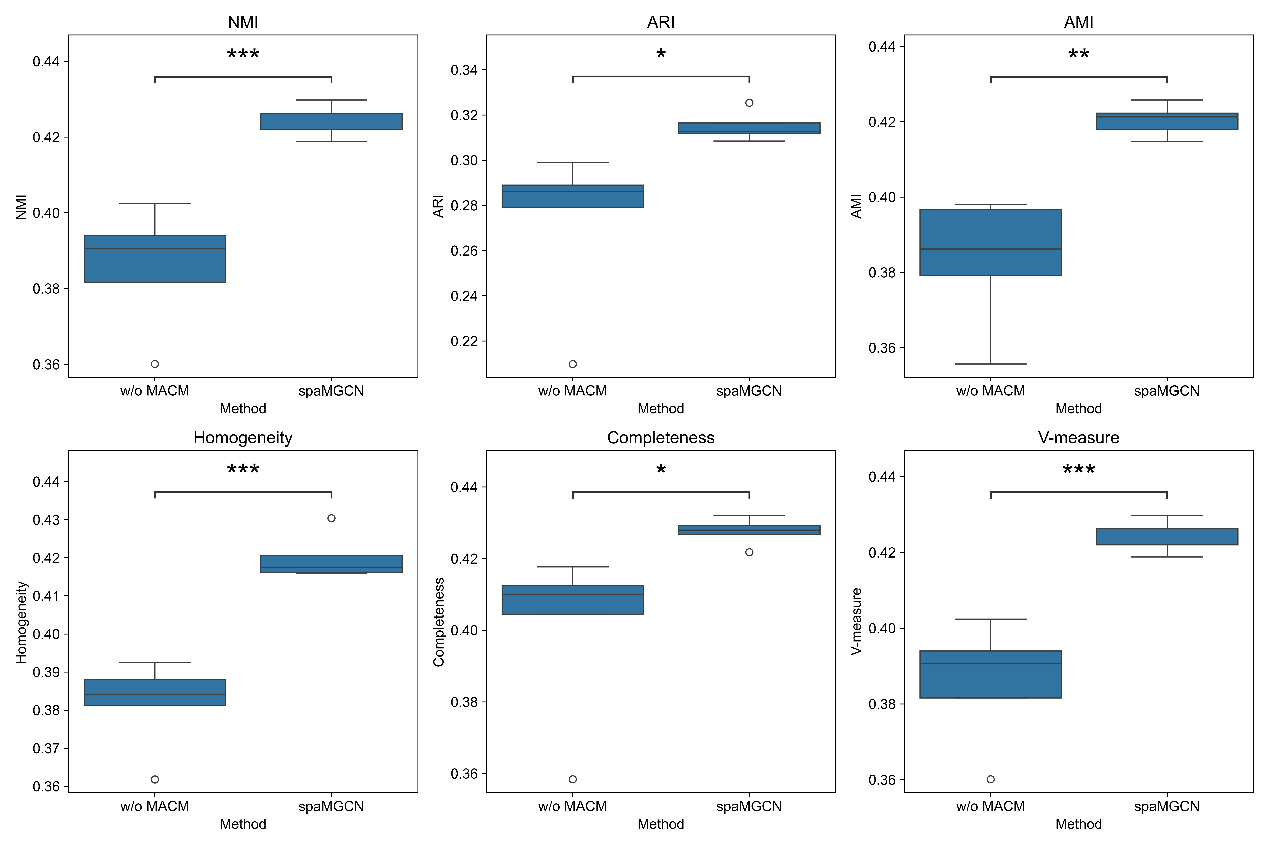


Fig. S28: Comparison of clustering performance between spaMGCN and its variant (w/o MACM) on the S3 dataset.
